# Anti-Parkinsonian Therapy: Strategies for Crossing the Blood–Brain Barrier and Nano-Biological Effects of Nanomaterials

**DOI:** 10.1007/s40820-022-00847-z

**Published:** 2022-04-15

**Authors:** Guowang Cheng, Yujing Liu, Rui Ma, Guopan Cheng, Yucheng Guan, Xiaojia Chen, Zhenfeng Wu, Tongkai Chen

**Affiliations:** 1 Key Laboratory of Modern Preparation of Traditional Chinese Medicine, Ministry of Education, Jiangxi University of Chinese Medicine, Nanchang, 330004 People’s Republic of China; 2 Science and Technology Innovation Center, Guangzhou University of Chinese Medicine, Guangzhou, 510405 People’s Republic of China; 3 State Key Laboratory of Quality Research in Chinese Medicine, Institute of Chinese Medical Sciences, University of Macau, Macau, 999078 People’s Republic of China

**Keywords:** Blood–brain barrier, Parkinson’s disease, Nasal delivery, Biomimetic drug delivery, Nano-biological effects

## Abstract

Parkinson’s disease (PD), a neurodegenerative disease that shows a high incidence in older individuals, is becoming increasingly prevalent. Unfortunately, there is no clinical cure for PD, and novel anti-PD drugs are therefore urgently required. However, the selective permeability of the blood–brain barrier (BBB) poses a huge challenge in the development of such drugs. Fortunately, through strategies based on the physiological characteristics of the BBB and other modifications, including enhancement of BBB permeability, nanotechnology can offer a solution to this problem and facilitate drug delivery across the BBB. Although nanomaterials are often used as carriers for PD treatment, their biological activity is ignored. Several studies in recent years have shown that nanomaterials can improve PD symptoms via their own nano-bio effects. In this review, we first summarize the physiological features of the BBB and then discuss the design of appropriate brain-targeted delivery nanoplatforms for PD treatment. Subsequently, we highlight the emerging strategies for crossing the BBB and the development of novel nanomaterials with anti-PD nano-biological effects. Finally, we discuss the current challenges in nanomaterial-based PD treatment and the future trends in this field. Our review emphasizes the clinical value of nanotechnology in PD treatment based on recent patents and could guide researchers working in this area in the future.

## Highlights

- Strategies for crossing the blood–brain barrier and the nano-biological effects of nanomaterials used for anti-Parkinsonian therapy are summarized.
- Patents related to nanotechnology-based anti-Parkinsonian therapy are reviewed, and the status of progress in this field are discussed.
- Current challenges in nanotechnology-based Parkinson’s disease treatment are discussed, with insights into the future trends in this field.

## Graphical abstract

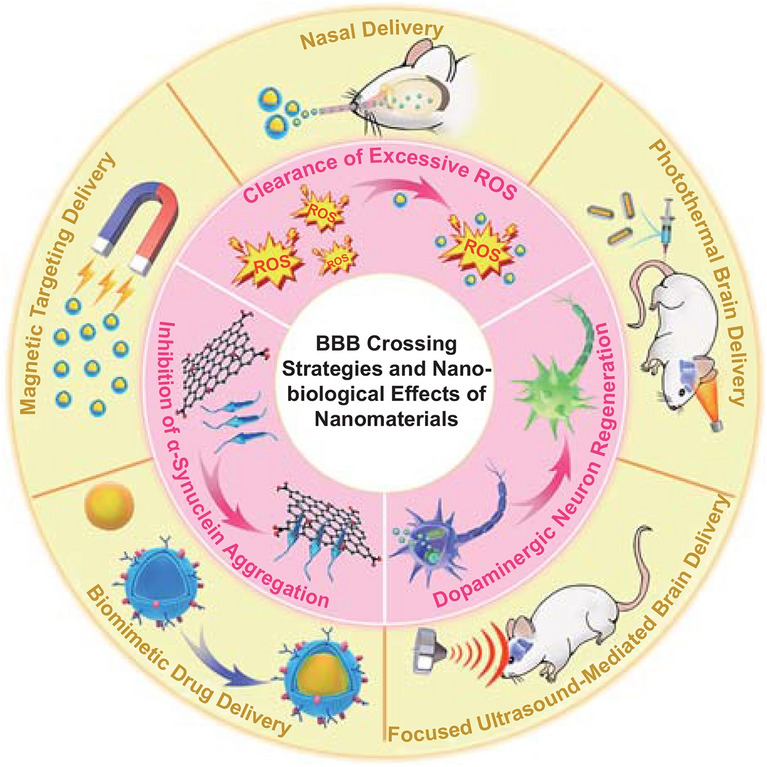

## Introduction

Population aging is inevitable and is accompanied by an increased prevalence of neurodegenerative diseases, including Parkinson’s disease (PD) [1]. In general, PD affects 2–3% of individuals aged ≥ 65 years, and by 2030, its prevalence is expected to rise by 50% [2]. PD was first described by James Parkinson in 1817 as a condition characterized by features like dyskinesia and a multitude of non-motor impairments, such as cognitive and autonomic dysfunction [3]. The main pathological manifestation of PD is the huge loss of dopaminergic neurons owing to the accumulation of reactive oxygen species (ROS) and the abnormal aggregation of the α-synuclein (α-syn) protein. The progression of PD is influenced by several factors, such as genetic predisposition, nerve inflammation, oxidative stress, and calcium homeostasis [4, 5]. Although the development of anti-PD drugs involves various critical aspects, the current clinical diagnosis of PD is mainly based on medical history, symptomatology and familial history, and it therefore has a low accuracy. The lack of an early-stage diagnostic biomarker is a particularly big challenge in the management of PD. The treatment strategies for PD primarily involve deep brain stimulation, exercise, and drug therapy. Deep brain stimulation therapy remains under development owing to several technical deficiencies that need to be overcome. Although factors such as exercise and environment have been shown to influence the progression of PD, no specific exercise regimen or method has been approved [6]. Therefore, chemotherapy is currently the dominant choice for anti-Parkinsonian therapy. The anti-PD drugs available in the market at present are listed in Table 1. Most drugs, such as dopaminergic, anti-muscarinic, and anti-glutamatergic medication [7, 8], only relieve dyskinesia and do not cure PD [9]. Therefore, advanced anti-PD drugs are urgently needed. Fortunately, based on the pathological characteristics of PD, novel therapies that target redox regulation, attenuation of α-syn misfolding and aggregation, and neuronal regeneration are being developed [10–12].

**Table 1:** List of Anti-Parkinsonian drugs, all with adverse effects on non-motor functions (data obtained from the Drugbank Database)

Name	Structure	Classification	Molecular weight	Solubility (mg mL^−1^)	Half-life (h)
Trihexyphenidyl		Anti-cholinergic	301.47	0.00314	3.3–4.1
Biperiden		Anti-cholinergic	311.46	0.00426	N/A
Benztropine		Anti-cholinergic	307.43	0.00121	36
Bromocriptine		Dopamine receptor agonist	654.56	0.0858	2–8
Lisuride		Dopamine receptor agonist	338.45	0.14	N/A
Pergolide		Dopamine receptor agonist	314.45	0.000584	27
Cabergoline		Dopamine receptor agonist	451.60	0.064	63–69
Ropinirole		Dopamine receptor agonist	260.37	0.353	6
Pramipexole		Dopamine receptor agonist	211.33	0.14	8.5–12
Rotigotine		Dopamine receptor agonist	315.48	0.00904	3
Apomorphine		Dopamine receptor agonist	267.32	0.51	0.83 (*i.v*)
Selegiline		Monoamine oxidase inhibitor	187.29	0.0254	1.2–2
Rasagiline		Monoamine oxidase inhibitor	171.24	0.0249	3
Entacapone		Catechol-o-methyltransferase inhibitor	305.29	0.0797	0.4–0.7
Tolcapone		Catechol-o-methyltransferase inhibitor	273.24	0.0569	2–3.5
Carbidopa		Dopa decarboxylase inhibitor	226.23	3.73	1.75
Benserazide		Peripheral decarboxylase inhibitor	257.25	5.15	1.5
Amantadine		Anti-glutamatergic	151.15	0.0846	10–14
Levodopa		N/A	197.19	3.3	2.3

The blood–brain barrier (BBB), a physiological barrier in the brain, poses the greatest challenge in the design of encephalopathy drugs. This barrier controls the movement of substances between the brain and the rest of the body and prevents nearly all large molecules and 98% of small molecules from entering the brain [13–15]. However, the protective nature of the BBB also limits the entry of anti-PD drugs, rendering them ineffective. Therefore, although many drugs, including matrine [16], curcumin [17] and other natural products derived from traditional Chinese medicines, have shown promising anti-PD potential in vitro, they have failed to exhibit corresponding effects in vivo. Great efforts have been made to devise strategies that enable drugs to cross the BBB. These include the use of special invasive injection routes to non-invasive strategies. Accordingly, the physiological functions of the BBB are gradually being understood. The brain, one of the most vital organs of the body, only accounts for 2% of body weight but requires 20% of the total energy of the body to maintain normal function [18]. The BBB contains various transporters and surface receptors that facilitate the delivery of substances across the BBB, allowing this high energy demand to be met. Moreover, lipophilic substances can easily enter the brain parenchyma through simple diffusion. Therefore, these physiological characteristics could be leveraged to design drugs that can overcome the BBB. Levodopa, a dopamine prodrug that is widely used in clinical settings, has considerable BBB permeability. However, it shows low potency due to a lack of targeting. Therefore, improving the brain-targeting efficiency of anti-PD drugs is another major challenge in PD treatment.

With the advancement of medical nanotechnology, the precision and efficiency of drug delivery are improving tremendously. This has brought new hope for the application of “old” drugs, such as herbal drugs and pnictogen-based drugs [19, 20]. Various nanomaterials have been shown to improve drug delivery to the brain [21, 22], and several BBB-compliant strategies have been developed based on its physiology. Since lipophilic substances can easily cross the BBB, several researchers have used lipophilic drug carriers to improve drug delivery to the brain. However, simple lipophilic carriers do not allow targeted drug delivery, and therefore, their efficiency remains low. For targeted drug delivery, ligands that specifically recognize surface receptors or transporters on the BBB are modified as drug carriers to facilitate receptor- or transporter-mediated BBB crossing. Although such strategies have improved targeted delivery, they lack efficiency, such that the drug enters the brain but fails to accumulate at the lesion site. Therefore, efficient nanomedicines for PD treatment require dual-targeting effects [23–26]. Recently, various BBB-crossing strategies have been developed, such as the bypassing of the BBB through nasal administration, development of nanomaterials that can permeate cell membranes, and ultrasound-mediated and photothermal drug delivery. The combined use of multiple strategies is expected to improve brain-targeted drug delivery and provide secondary targeted therapy for PD. Further, such anti-PD drugs also have fewer adverse effects and improved clinical efficiency [27, 28].

Currently, nanomaterials are not very popular as drug carriers owing to biocompatibility issues; however, they have certain biological effects, which cannot be ignored. Notably, several nanomaterials possess physiological activities that can be exploited for clinical applications [29, 30]. Here, we review various nanomaterials that can be used to treat PD via nano-biological effects. For instance, some nanomaterials, such as black phosphorus nanosheets [31], carboxyfullerenes [32], and copper-based nanoclusters [33], contain redox-capable components that remove excessive ROS from the PD lesion site and thereby improve the survival of dopaminergic neurons. Such nanomaterials with therapeutic activity can directly aid in PD treatment without the need for drug loading. In this review, we first provide a brief overview of the BBB and then summarize ideas for designing nanoplatforms capable of crossing the BBB. Moreover, we elaborate on various BBB-crossing strategies and their applications in PD treatment. Subsequently, we discuss the nanomaterials with anti-PD nano-bio effects. Furthermore, to emphasize the clinical value of nanoplatforms and the trends of their development, we present the relevant patents over recent years. Finally, we review the current challenges in the field and propose potential solutions that can be used to overcome them.

## Physiology of the BBB and Physiopathology of PD

**Fig. 1:**
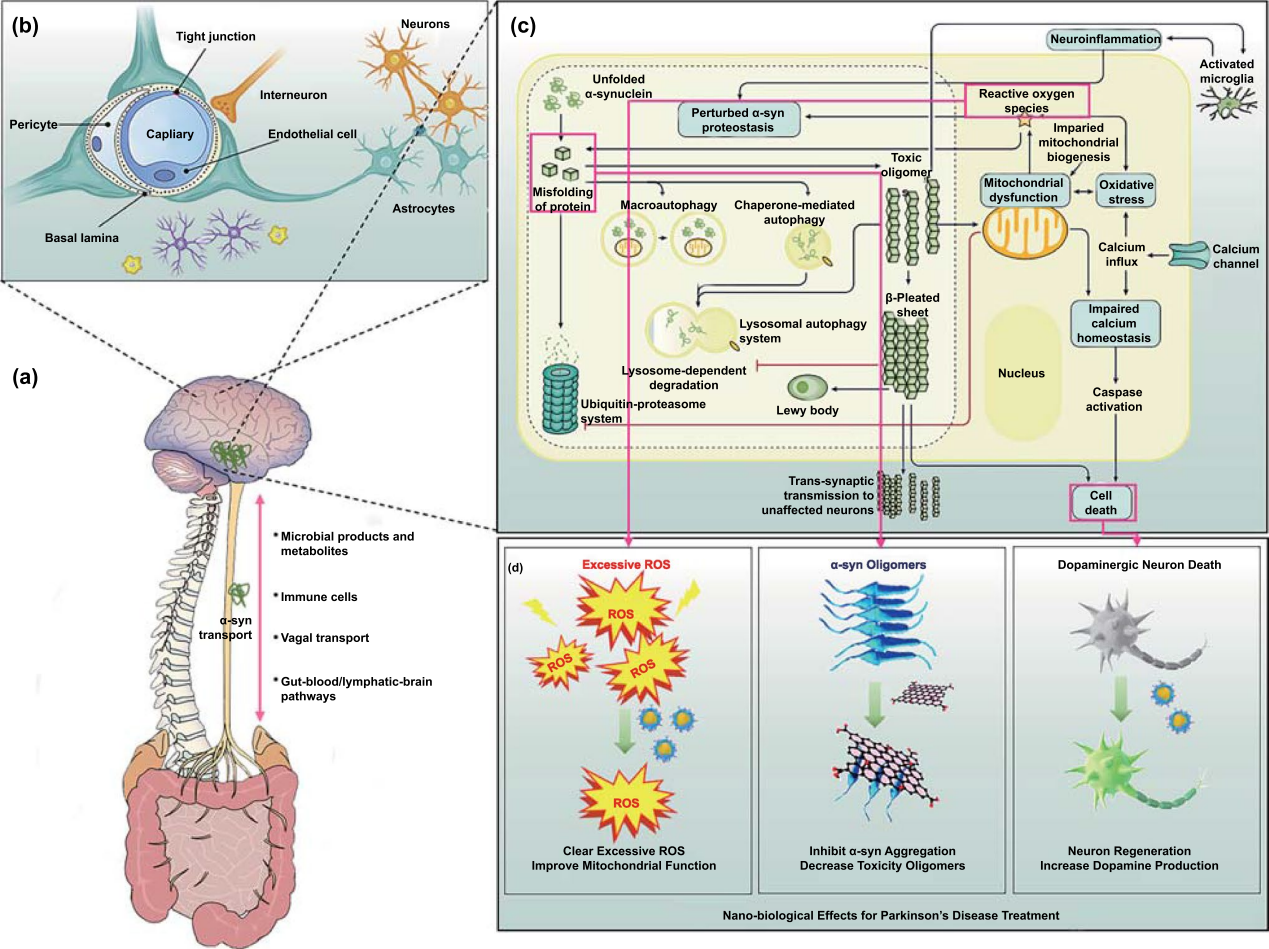
Pathology of PD and nano-bio effects useful for its treatment. **a** The gut microbiota–brain axis that promotes the transport of α-syn from the brain to the gut. The brain-gut communication pathways are of four types: (1) microbial products and metabolites that directly enter the brain; (2) the microbiome, which regulates the immune systems and indirectly influences the CNS; (3) signals sent to the CNS through vagal nerve terminals; (4) gut–blood/lymphatic–brain pathways, through which the microbiome or its metabolites can directly enter the brain. Adapted with permission from Ref. [40]. Copyright 2021 Springe Nature. **b** The physiological structure of the BBB, which stop drug enter the CNS. Adapted with permission from Ref. [45]. Copyright 2019 Elsevier B.V. **c** Schematic diagram depicting interactions between major molecular pathways that are implicated in the pathogenesis of Parkinson disease. Adapted with permission from Ref. [4]. Copyright 2017 Springe Nature. **d** Nano-bio effects of clearing excessive ROS, inhibiting α-syn aggregation, and promoting neuron regeneration for PD treatment

Understanding the physiological characteristics of the BBB and the molecular mechanisms of PD physiopathology is undoubtedly of great significance for PD treatment. In this section, we will illustrate and briefly summarize the physiological characteristics of the BBB and the molecular mechanisms involved in PD (Fig. 1).

### Physiology of the Blood–Brain Barrier

The BBB, a vital boundary between the brain microenvironment and the external environment, maintains brain homeostasis by controlling the exchange of substances between the brain and blood. It is composed of endothelial cells (ECs), pericytes, astrocytes, and a basement membrane. The closely packed ECs and the tight junctions (TJs) between adjacent ECs offer ultrahigh transendothelial resistance [34]. ECs and TJs together regulate the permeability of the BBB; therefore, many researchers have used ECs to construct in vitro models of the BBB [35]. The basement membrane consists of various deposited proteins secreted by ECs, astrocytes, and pericytes. The non-cellular components of the BBB regulate the stability of the microvascular system [36]. Pericytes, located on the periphery of ECs, share the basement membrane with these cells. Pericytes and ECs communicate with each other via N-cadherin and connexins. Pericytes maintain the integrity of the BBB by regulating the diameter of capillaries and phagocytosing toxic metabolites [37]. Moreover, the polarized cell protrusions of astrocytes cover neuronal protrusions and blood vessels, creating a transmission network between blood vessels and neuronal circuits [38]. Dynamic balance among the respective components is necessary for BBB homeostasis.

### Physiopathology of PD

The causes of PD are complicated. Race, sex, genes, environment, and lifestyle all have an impact on the incidence of PD, and the intricate relationships between these factors remain to be clarified. Currently, basic research on PD mainly focuses on the molecular mechanisms of its pathological development. Here, we briefly summarize the mechanisms involved in the development of this disease.

Although the exact mechanisms mediating the onset of PD are unclear, PD development is mainly promoted by neurotoxic oligomers consisting of α-synuclein, excessive ROS production, neuroinflammation and dopaminergic neuron loss. The removal of soluble α-synuclein is mediated by the ubiquitin protease system and lysosomal autophagy [4, 8]. However, when the soluble monomers show abnormal aggregation, insoluble α-synuclein gets converted into fibrils, mitochondrial function gets destroyed, ROS production increases, and oxidative stress occurs. In turn, mitochondrial dysfunction inhibits the ubiquitin protease system and lysosomal autophagy, further accelerating the production of neurotoxic α-syn oligomers. In addition, mitochondrial dysfunction also causes calcium influx, aggravates oxidative stress, and induces neuronal apoptosis. The α-syn oligomers also have prion-like diffusion characteristics and can spread to other brain regions through axons [39]. Therefore, α-syn aggregates can be transported to microglia, where they induce M1 polarization and promote the release of inflammatory factors, thus causing neuroinflammation. This further interferes with the protein homeostasis of soluble α-syn and aggravates the formation of neurotoxic aggregates.

It is worth mentioning that the non-motor disorders of PD include gastrointestinal problems, suggesting that disease development in PD is also related to the gastrointestinal tract. Recently, studies have shown that the central nervous system (CNS) is related to the gastrointestinal tract through the “brain-gut axis,” which originates from the brainstem and spinal cord and innervates the intestines through the vagus nerve, splanchnic nerve, mesenteric nerve and pelvic spinal cord nerve [40, 41]. The communication between the gastrointestinal tract and the CNS is bidirectional [42]. The CNS can communicate with the enteric nervous system via autonomic activity. For example, phosphorylated α-syn can be transmitted from the intestines to the brain through the vagus nerve [43]. In turn, the intestinal tract can communicate with the CNS or enteric nervous system through secreted molecules, such as lipopolysaccharides, SCFAs, branched chain amino acids, neurotransmitters, and serotonin derived from the intestinal flora. Moreover, under the pathological conditions of PD, immune cell Toll-like receptor TLR4/2 can be activated by intestinal microbial metabolites, intensifying neuroinflammation. In an important study, Sampson et al*.* transferred the intestinal microbiome of PD patients into mice and found that the mice developed motor dysfunction after treatment [44]. Therefore, the treatment of PD can also start from the gastrointestinal tract. The removal of peripheral α-syn and the regulation of the gastrointestinal microbiome could become new strategies for PD treatment.

## Nanoplatforms Designed to Cross the BBB

Nanoplatforms designed for brain drug delivery as well as their metabolites should be non-toxic or have low toxicity. Further, they should allow the complete realization of the drug’s pharmaceutical activity without hampering its efficacy. Importantly, the nanoplatforms must be stable in the blood and should prevent self-degradation-mediated early drug leakage to ensure that the drug is released only in the brain. There are various nanoplatforms, and these can be divided into three categories, all of which have their own advantages: organic nanocarriers, inorganic nanocarriers, and organic–inorganic hybrid nanocarriers [46]. The biocompatibility of organic nanocarriers is relatively high, while inorganic nanocarriers usually show better light absorption capacity and a few of them also possess magnetic properties (e.g., iron oxide nanoparticles). In contrast, organic–inorganic hybrid nanocarriers have extremely high editability and can be easily manipulated to achieve responsive drug release. However, their drug loading capacity, morphological features, surface potential, and connected modifiers affect the efficiency of brain-targeted delivery.

Drug loading capacity is an important indicator of the quality of nano-drug delivery systems. If the drug loading capacity is low, the therapeutic window is not met, which influences drug efficacy and compliance. Thus, both the drug loading content and efficiency of nanoplatforms are evaluated. The drug loading content refers to the mass ratio of the drug loaded onto the nanoplatform and mainly depends on the structural, physical, and chemical properties of the nanoplatform. In contrast, the drug loading efficiency refers to the ratio between the drug loaded onto the nanoplatform and the drug input during preparation, and it depends on the interaction (s) between the drug and the nanoplatform [47]. Notably, it is more challenging to achieve a high drug loading efficiency than to achieve a high drug loading content, and in most cases, the drug loading efficiency remains lower than 10% [48]. Recent studies suggest that nanoplatforms with higher specific surface areas, such as porous silica nanoparticles (NPs) or two-dimensional nanomaterials, offer a better drug loading capacity. The drug loading content of black phosphorous nanosheets with a “zig” structure can reach up to 950% [49]. In general, nanoplatforms with a higher drug loading capacity offer better drug delivery efficiency. The particle size of the nanoplatform is another important factor affecting its cellular uptake and BBB-crossing efficiency [50]. Oshra et al*.* designed various NPs of different sizes (20, 50, and 70 nm) to evaluate their capability of crossing the BBB [51] and found that small-size NPs (20 nm) showed the best crossing activity. Ultrasmall carbon dots with higher cell membrane permeability can easily cross the BBB without requiring any modification [52]. However, a nanoplatform sized < 5 nm is easily excreted by glomeruli. Therefore, the best particle size is thought to range from 5 to 200 nm [53]. Apart from size, the shape of the nanoplatform also influences its cellular uptake [54]. Recently, a study based on coarse-grained molecular dynamics simulation revealed the significance of shape in the cellular uptake of NPs [55]. Oblate NPs, with a larger contact surface area, showed a higher uptake efficiency than spherical and prolate NPs. In another study, Kolhar et al*.* too found that rod-shaped nanostructures with larger contact surfaces were more easily taken up by the brain endothelium than were spherical NPs [56]. Therefore, a nanoplatform with a larger contact surface area is believed to be better for drug delivery to the brain parenchyma.

The surface charge of the nanoplatform also plays a major role in targeted drug delivery via the adsorptive-mediated transcytosis (AMT) pathway. Byeon et al*.* prepared c/m-HSA (zeta potential -12.0 ± 0.3 mV) and HAS (zeta potential -34.6 ± 1.4 mV) nanoplatforms via differential surface modifications and tested them in vivo using fluorescent labeling. They found that the fluorescence signal for c/m-HSA NPs in the brain was significantly higher than that for HSA NPs, suggesting that a higher zeta potential is conducive for nanoplatform delivery across the BBB [57]. Moreover, a higher zeta potential also helps NPs bypass lysosomal degradation in endothelial cells during the traversing of the BBB.

Nanoplatforms for brain drug delivery can be classified based on the type of targeting: active vs. passive. In the former system, delivery efficiency to the brain depends on systemic circulation. However, such nanoplatforms are limited by elimination via the kidneys, reticuloendothelial system, and immune system. Polyethylene glycol (PEG)-based nanoplatforms can avoid rapid renal elimination and also effectively inhibit the immune system, thereby showing improved systemic circulation and biocompatibility [58]. However, the long-term usage of PEGylated drugs adversely affects the immune system [59]. Therefore, new polymer materials such as hyaluronic acid [60] and sulfobetaine [61] have been recommended as replacements. Apart from systemic circulation, BBB crossing is also crucial for drug delivery to the brain. Normal brain function requires diverse and large amounts of nutrients, and there are therefore multiple transport pathways across the BBB. Hence, brain delivery can be enhanced by leveraging the physiological characteristics of the BBB. As discussed previously, BBB function is primarily ascribed to ECs, but the lipophilicity of ECs allows the simple diffusion of lipophilic substances. Therefore, lipophilic nanoplatforms, such as liposomes and polymeric NPs, are suitable carriers for brain drug delivery. In addition, there are multiple hormone-regulated surface transporters on the BBB that facilitate the transport of certain nutrients in a process called carrier-mediated transcytosis (CMT) [62]. The BBB also has surface receptors for other substances, including transferrin, insulin, leptin, lipoprotein, diphtheria toxin, folate, vasopressin, and glycation end products [28] that specifically recognize large endogenous substances and transport them via receptor-mediated transcytosis (RMT). For efficient brain-targeted delivery, nanoplatforms should be modified with a ligand. The required modifications can be divided into three types: saccharide, protein, and peptide modifications. Modified nanoplatforms provide brain-targeted drug delivery by exploiting the CMT and RMT pathways. It must be noted that in addition to CMT and RMT ligands, cationic or amphiphilic substances can also be used as surface attachments to increase the positive charge on nanoplatforms and allow electrostatic adsorption on endothelial cells as well as the use of the AMT pathway [63]. Unfortunately, the AMT strategy provides poor brain delivery efficiency due to its non-specificity and requires a complex preparation process; this strategy is thus seldom applied.

Although brain-targeted nanoplatforms prepared based on the physiological characteristics of the BBB show a higher brain aggregation rate than do free drugs, they mainly aggregate in organs such as the liver and kidneys, indicating that targeting efficiency to the brain needs to be improved. To improve brain-targeted delivery, various strategies that are not solely dependent on physiological characteristics have been developed in recent years. These strategies are of three types: (1) Use physiological characteristics to bypass the BBB; (2) Coat nanoplatforms with endogenous biofilms to cheat the BBB; and 3. Increase BBB permeability via external treatments (external magnetic field, near-infrared-mediated photothermal effect, and focused ultrasound). All these strategies can improve the BBB-crossing capability of drugs while ensuring safety. The combination of multiple strategies is expected to provide more efficient brain delivery and could be promising for achieving the secondary targeting of brain lesions.

**Fig. 2:**
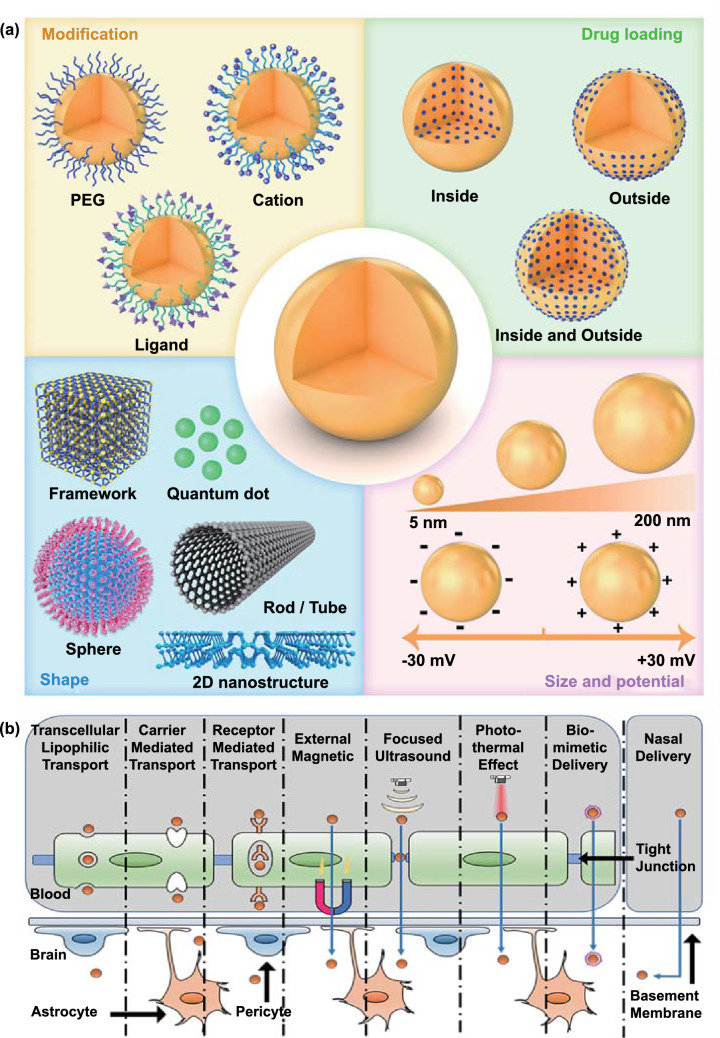
Design strategies for nanoplatforms that can cross the BBB (**a)**, and transport pathways used in PD treatment (**b)**

In summary, nanoplatforms designed for brain delivery must have a suitable appearance, zeta potential, biocompatibility, and BBB-crossing capability (Fig. 2a). Moreover, special ligands and strategies may sometimes be required to improve brain delivery efficiency and achieve secondary targeting in cases where the disease is localized to a particular part of the brain. These strategies are summarized in Fig. 2b.

## Applications of BBB-Crossing Strategies for PD Treatment

Initially, in order to enable anti-PD drugs to exert their effects, only invasive delivery strategies, including intracranial injection, intraventricular injection, and convection enhancement, were used [64]. Such strategies have severe adverse effects and also require resources such as surgical personnel and facilities. Although the subsequent development of intrathecal injections has partly mitigated this issue, the procedure remains invasive and compliance remains poor [65]. Currently, the most common clinical strategy for drug delivery to the brain is the conversion of the drug into a prodrug, such as levodopa, that can penetrate the BBB. However, this strategy is not sufficient to eliminate the challenges associated with the drug’s short half-life and also does not provide targeted delivery [66]. In contrast, non-invasive nanoplatform-based drug delivery strategies are more suitable for anti-PD drug delivery. In recent years, a considerable amount of research has been devoted toward understanding the advantages of nanoplatforms in crossing the BBB and their potential application in PD treatment (Table 2). In this section, we will discuss these non-invasive delivery strategies and their applications in PD treatment in detail.

**Table 2:** Emerging nanoplatform-based BBB-crossing strategies for PD treatment

Nanoplatform	Cargos	Size (nm)	Zeta potential (mV)	Drug loading (DL)/entrapment efficiency (EE) (%)	Establish PD model	Crossing BBB mechanism	Administration route	References
Nanoparticle	Levodopa	254.1	6.87 ± 1.10	56 ± 0.14 (EE)	MPTP	Transcellular lipophilic transport	Intravenous injection	[67]
Nanoparticle	Epigallocatechin-3-gallate	36.3 ± 2.4	5.6 ± 1.7	83.4 ± 3.8 (EE)	Transgenic mice	Transcellular lipophilic transport	Intravenous injection	[68]
Nanocrystal	Resveratrol	222.54 ± 1.66	− 9.41 ± 0.37	21.74 (DL)	MPTP	Transcellular lipophilic transport	Oral	[69]
Nanocrystal	Ginkgolide B	83.48 ± 1.77	− 19.25 ± 1.04	44.44 (DL)	MPTP	Transcellular lipophilic transport	Oral	[70]
Nanocrystal	Schisantherin A	160.33 ± 2.08	N/A	33.3 (DL)	MPTP	Transcellular lipophilic transport	Oral	[71]
Nanocrystal	Puerarin	83.05 ± 1.96	− 22.41 ± 1.19	72.7 (DL)	MPTP	Transcellular lipophilic transport	Oral	[72]
Micelle	Baicalein	40.61 ± 1.17	19.59 ± 1.84	7.07 ± 0.27 (DL)	MPTP	Transcellular lipophilic transport	Oral	[73]
Nanoparticle	Ropinirole	193.2 ± 2.1	− 30.5 ± 1.5	8.4 ± 0.3 (DL)	Haloperidol	Transcellular lipophilic transport	Oral	[74]
Nanoparticle	Curcumin and piperine	60	− 30.9 ± 0.88	65 (EE)	Rotenone	Transcellular lipophilic transport	Oral	[75]
Nanoparticle	Puerarin	88.36 ± 1.67	− 18.85 ± 2.76	42.97 ± 1.58 (DL)	MPTP	Transcellular lipophilic transport	Oral	[76]
Nanoparticle	Ropinirole	152.2 ± 3.1	14.25 ± 0.43	74.8 ± 8.2 (EE)	Rotenone	Transcellular lipophilic transport	Intraperitoneal injection	[77]
Nanoparticle	Plasmid DNA	5	− 22.5	N/A	MPTP	Transcellular lipophilic transport	Intraperitoneal injection	[78]
Nanoparticle	Melatonin	79 ± 13	− 24.6	45.4 ± 1.9 (DL)	Rotenone	Transcellular lipophilic transport	Intravenous injection	[79]
Nanoparticle	Metformin	80 ± 5	− 22 ± 0.6	45.2 ± 5 (DL)	Rotenone	Transcellular lipophilic transport	Intraperitoneal injection	[80]
Nanoparticle	Selegiline	286.1 ± 4.23	− 29.11	N/A	Reserpine	Transcellular lipophilic transport	Intraperitoneal injection	[81]
Nanoparticle	Schisantherin A	70.6 ± 2.2	− 24.7 ± 3.5	28.0 ± 0.8 (DL)	MPTP	Transcellular lipophilic transport	Oral	[82]
Nanoparticle	Levodopa	52.2	N/A	N/A	MPTP	Transcellular lipophilic transport	Intraperitoneal injection	[83]
Nanoparticle	Vascular endothelial growth factor	220.1 ± 11.2	− 18.6 ± 4.2	N/A	6-OHDA	Transcellular lipophilic transport	Intravenous injection	[84]
Nanoemulsion	Rutin	18	− 41	N/A	Haloperidol	Transcellular lipophilic transport	Oral	[85]
Nanoemulsion	Coenzyme Q10	20.05–2.53	− 24.40–0.16	N/A	Haloperidol	Transcellular lipophilic transport	Oral	[86]
Nanoemulgel	Selegiline	183.4 ± 0.35	N/A	N/A	6-OHDA	Transcellular lipophilic transport	Oral	[87]
Nanoparticle	pDNA	200	− 15	62 (EE)	MPTP	Carrier-mediated transport	Intraperitoneal injection	[88]
Nanoparticle	pDNA	290.400	N/A	37 (DL)	MPTP	Receptor-mediated transport	Intraperitoneal injection	[89]
Nanoparticle	microRNA	162 ± 1.25	5.4 ± 1.45	N/A	MPTP	Receptor-mediated transport	Intraperitoneal injection	[90]
Nanoparticle	Deferoxamine	168.8 ± 1.9	− 27.40 ± 0.71	9.2 ± 0.9 (DL)	MPTP	Receptor-mediated transport	Intravenous injection	[91]
Liposome	Dopamine derivative	134.7 ± 2.66	− 13.5 ± 0.37	6.15 ± 0.17 (DL)	6-OHDA	Receptor-mediated transport	Intravenous injection	[92]
Liposome	Dopamine	100	− 33.7	N/A	6-OHDA	Receptor-mediated transport	Intraperitoneal injection	[93]
Nanoparticle	4,4′-Dimethoxychalcone	~ 100	4.5	45.2 ± 2.8 (EE)	MPTP	Receptor-mediated transport	Intravenous injected	[94]
Nanoparticle	Epigallocatechin gallate	80	− 0.2–0.7	N/A	Transgenic mice	Receptor-mediated transport	Intravenous injection	[95]
Nanoparticle	siRNA	153.9 ± 4.08	− 41.86 ± 1.048	N/A	MPTP	Receptor-mediated transport	Intravenous injection	[96]
Nanotube	Dopamine	< 200	N/A	N/A	6-OHDA	Receptor-mediated transport	Intraperitoneal injection	[97]
Nanoparticle	Selegiline	341.6 ± 56.91	13.4 ± 0.04	92.20 ± 7.15 (EE)	Rotenone	Bypass the BBB	Intranasal injection	[98]
Nanoparticle	Rotigotine	122.0 ± 19.3	− 21.28 ± 2.15	N/A	N/A	Bypass the BBB	Intranasal injection	[99]
Nanocrystal	Paeoniflorin	152.4 ± 3.6	− 23.2 ± 0.529	N/A	MPP^+^	Bypass the BBB	Intranasal injection	[100]
Chitosan-coated nanoparticle	Rotigotine	75.37 ± 3.37	25.53 ± 0.45	96.08 ± 0.01 (EE)	6-OHDA	Bypass the BBB	Intranasal injection	[101]
Nanoemulsion	Selegiline	61.43 ± 4.10	-34	N/A	Haloperidol	Bypass the BBB	Intranasal injection	[102]
Nanoemulsion	Vitamin E	38.70 ± 3.11	− 27.4 ± 0.14	N/A	6-OHDA	Bypass the BBB	Intranasal injection	[103]
Nanoemulsion	L-DOPA	383.7 ± 66.94	− 20.8 ± 3.63	50.47 (EE)	MPTP	Bypass the BBB	Intranasal injection	[104]
Nanostructured lipid carrier	Glial cell-derived neurotrophic factor	205.9 ± 6.3	21.9 ± 1.8	87.66 (EE)	MPTP	Bypass the BBB	Intranasal injection	[105]
Lipid/polymeric nanoparticle	Geraniol/ursodeoxycholic acid conjugate	121 ± 8.4/181 ± 5.9	− 26.7 ± 6.5	12.1 ± 1.4 (DL)	N/A	Bypass the BBB	Intranasal injection	[106]
*in*Pentasome	Pentamidine	300.7 ± 17.2	39.0 ± 1.1	24 (EE)	MPTP	Bypass the BBB	Intranasal injection	[107]
Chitosan-coated nanostructure lipid carrier	Glial cell-derived neurotrophic factor	136.70 ± 14.14	30	98.10 ± 0.36 (EE)	6-OHDA	Bypass the BBB	Intranasal injection	[108]
Curcumin analog-based nanoscavenger	Curcumin	131.1	− 38.6	26.95 (DL)	MPTP	Bypass the BBB	Intranasal injection	[109]
Nanoparticle	Dopamine	175.3 ± 9.6	− 15.7 ± 0.86	25.43 ± 5.32 (EE)	6-DOPA	Bypass the BBB	Intranasal injection	[110]
Hydrogel	Ropinirole	N/A	N/A	80.18 (EE)	N/A	Bypass the BBB	Intranasal injection	[111]
Hydrogel	Curcumin	N/A	N/A	N/A	MPTP	Bypass the BBB	Intranasal injection	[112]
Nanoscale coordination polymer	Dopamine	81 ± 4.0	−8.32 ± 0.25	52.5 ± 7.2% (EE)	6-OHDA	Bypass the BBB	Intranasal injection	[113]
Exosome	Curcumin and iron oxide nanoparticles	135.9–194.9	− 7.23	77.50% for iron oxide nanoparticles and 75.53% for curcumin (EE)	MPTP	Bypass the BBB	Intranasal injection	[114]
Exosome	DNA aptamer	100	N/A	26 (EE)	PFFs	Biofilm camouflage	Intraperitoneal injection	[115]
Exosome	Dopamine	70–100	N/A	15 ± 0.22 (DL)	6-OHDA	Biofilm camouflage	Intravenous injection	[116]
Exosome	Curcumin and siRNA	141.0	7.05	70 (EE)	MPTP	Biofilm camouflage	Intravenous injection	[117]
Fe_3_O_4_-modified liposome	Nimodipine	164 ± 3	N/A	93.56	6-OHDA	Magnetic	Intraperitoneal injection	[118]
Fe_3_O_4_-modified liposome	Resveratrol	163	N/A	86.94 ± 1.94 (EE)	6-OHDA	Magnetic	Intraperitoneal injection	[119]
Black phosphorus nanosheet	Paeoniflorin	203.1	5.15	N/A	MPTP	Photothermal	Intravenous injection	[120]
Mesoporous silica-encapsulated gold nanorod	Quercetin	15.7 × 91.3	N/A	13.5 (DL)	MPTP	Photothermal	Intravenous injection	[121]
Zeolitic imidazolate framework-8	Prussian blue and quercetin	143	N/A	61.3 (EE)	MPTP	Photothermal	Intravenous injection	[122]
Liposome	Recombinant human fibroblast growth factor-20	68.1 ± 2.1	N/A	N/A	6-DOPA	Focus ultrasound-mediated	Intravenous injection	[123]
Nanoparticle	Glial cell-derived neurotrophic factor	50 ± 3	1.5 ± 0.2	N/A	6-DOPA	Focus ultrasound-mediated	Intravenous injection	[124]
Polysorbate 80-modified cerasome	Curcumin	110.43 ± 6.59	−25.0 ± 0.9	86 ± 1.25 (EE)	MPTP	Focus ultrasound-mediated	Intravenous injection	[125]
Lipid nanomicrobubble	Glial cell -derived neurotrophic factor	100 ~ 4200	2.3 ± 1.9	N/A	MPTP	Focus ultrasound-mediated	Intravenous injection	[126]
Lipid nanomicrobubble	Nuclear factor E2-related factor 2	313.5 ± 47.68	14.8 ± 3.99	N/A	6-OHDA	Focus ultrasound-mediated	Intramuscular injection	[127]
Cell membrane-coated nanoparticles	Quercetin	78.8	−35.2	17.6 (DL)	MPTP	Focus ultrasound-mediated	Intravenous injection	[128]

### Biological Strategies

As mentioned before, the body ensures that the substances required for the normal physiological activities of the brain across the BBB. This material exchange process provides inspiration for brain delivery. In addition, under certain pathological conditions, certain cells can also enter the brain parenchyma in order to maintain the relative homeostasis of the brain microenvironment. This physiological activity also provides opportunities for brain delivery. In this part, strategies for crossing the BBB based on various physiological functions will be discussed.

#### Transcellular Lipophilic Transport

Many nanoplatforms, including liposomes and nano-lipid carriers, are developed by simulating the characteristics of biological membranes. Such nanoplatforms are similar to cell membranes and are composed of phospholipids, which not only guarantees their biocompatibility but also makes them highly lipophilic. Owing to such properties, these nanoplatforms have ideal BBB permeability and are thus excellent for drug delivery to the brain [129]. Other polymer-based nanoplatforms, such as micelles [90], nanoemulsions [86], polymeric NPs [79], and nanocrystals [71], can be directly transported into the brain via intracellular pathways. The mechanism underlying this transport may be related to enhanced endocytosis of these lipophilic copolymers or surfactants by ECs [130]. These nanoplatforms have advantages such as easy preparation, low cost, and stability. In recent years, they have been used to improve the delivery of puerarin [72], ginkgolide [70], and other drugs with anti-PD potential that cannot cross the BBB.

Although the lipophilicity of nanoplatforms can significantly enhance their accumulation in the brain, a comparison of blood and brain pharmacokinetic parameters reveals little difference between free drugs and lipophilic NPs, indicating that the efficiency of drug delivery to the brain is low. For example, Chen et al*.* loaded puerarin onto star-shaped PLGA NPs to develop an oral nanomedicine [76]. Through pharmacokinetic analysis of brain tissue, they found that the nanocarrier increased the amount of puerarin in the brain only 6.46-fold, although blood pharmacokinetic analysis revealed a more significant improvement in the systemic circulation of puerarin (tenfold), indicating that the efficiency of delivery to the brain was lower in the nanomedicine group than in the free drug group. The reason for this phenomenon was the lack of nanocarrier targeting to the brain and drug release during systemic circulation. Therefore, using a targeting ligand on a lipophilic nanoplatform can allow quicker drug entry into the brain parenchyma, not only enhancing the brain-targeting efficiency of the nanoplatform but also reducing the distribution and release of drugs in other organs. Such a modification could be of great significance for improving the efficacy of anti-PD drugs.

#### Carrier-Mediated Transport

**Fig. 3:**
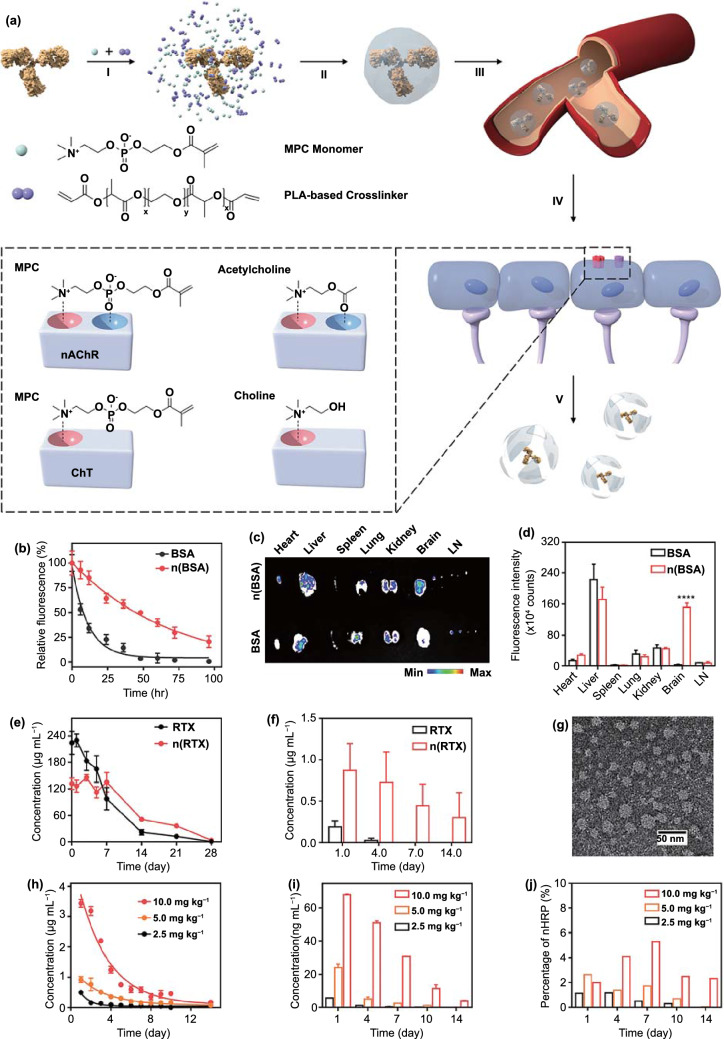
CMT strategy for BBB crossing. **a** Schematic illustration of the design of the bioinspired delivery system for effective delivery of therapeutic proteins to the CNS. **b** Pharmacokinetic profiles of mice after intravenous injection of 1 mg mL-1 native BSA or n(BSA) labeled with TAMRA. **c** Ex vivo images and **d** normalized fluorescence intensity of dissected tissues including brain, heart, liver, spleen, lung, kidney, and lymph node tissues, from mice treated with TAMRA-labeled BSA and n(BSA). The mice were perfused with PBS and organs were harvested 24 h post-intravenous injection. **e** RTX plasma and **f** CSF concentration in mice intravenously injected with 5 mg kg^−1^ of native RTX or n(RTX), measured by ELISA. **g** TEM images of CSF obtained from rhesus macaques 24 h after the intravenous administration of 10 mg kg-1 n(HRP). **h** Plasma and **i** CSF concentrations of n(HRP) and the **j** the ratio of CSF versus plasma concentration of n(HRP) in rhesus macaques after the intravenous administration of 2.5, 5.0, and 10 mg kg^−1^ of n(HRP). Adapted with permission from Ref. [132]. Copyright 2019 WILEY–VCH Verlag GmbH & Co. KgaA, Weinheim

In order to transport substances such as carbohydrates, amino acids, monosaccharides, fatty acids, hormones, calcium ions, and choline into the brain, the various transporters present on the surface of ECs undergo conformational changes when they recognize their substrates, such as carbohydrates, amino acids, monosaccharides, fatty acids, hormones, calcium ions, and choline [131]. Adding such a substrate to the surface of nanoplatforms can enhance their BBB-crossing capability. Recently, Lu et al*.* developed a polymer that can recognize choline and acetylcholine and could thus improve brain-targeted delivery (Fig. 3a) [132]. To confirm the brain delivery efficiency, carboxytetramethylrhodamine (TAMRA)-labeled bovine serum albumin (BSA) was loaded onto the polymer-based carrier, and the pharmacokinetics and biodistribution of nBSA and BSA were tested. The results of pharmacokinetic analysis showed that nBSA had a long circulation period (Fig. 3b), likely because the adsorption of serum proteins was prevented by the polymer shell, thereby reducing clearance by macrophages. Moreover, nBSA exhibited significantly stronger brain aggregation than free BSA (Fig. 3c-d). In this study, they also used the carrier to load rituximab (nRTX) and horseradish peroxidase (nHRP) and confirmed the brain-targeting efficiency of the two molecules. As shown in Fig. 3e-g, nRTX had better systemic circulation than RTX, and after intravenous injection, the levels of nRTX in the cerebrospinal fluid were also significantly higher than those of free RTX. Moreover, after high-dose administration, nHRP showed considerable brain aggregation (Fig. 3h-j). These results prove that this polymer, based on the CMT strategy, can cross the BBB and thus has excellent application prospects in the treatment of neurological diseases, including PD. More importantly, several of these substrates also have their own physiological activities. Thus, when designing anti-PD nanomedicines based on the CMT strategy, substrates with a PD-alleviating effect can be selected as modifiers. For example, the transporter substrate docosahexaenoic acid (DHA) can improve cognitive function and memory in patients [133]; thus, DHA-modified nanoplatforms could provide synergistic treatment along with other drugs. There has been some preliminary research on such strategies. For example, Guan et al*.* modified plasmid DNA-loaded nanocarriers with DHA to design a nanomedicine for dyskinesia and observed improvements in non-motor dysfunction in PD models [88].

In general, the modification of anti-PD nanoplatforms with a transporter substrate not only provides a certain amount of brain-targeting efficiency but can also allow combination treatment of PD with modifiers and drugs. However, this strategy also has many insurmountable defects. First, CMT is concentration gradient-dependent; second, the CMT transporter is not specifically expressed in the brain and is also present in other tissues; and third, the number of transporters is limited. Owing to these shortcomings, the CMT strategy alone cannot be used to achieve extremely high brain-targeted drug delivery efficiency via nanoplatforms, and its combination with other strategies is expected to mitigate these problems.

#### Receptor-Mediated Transport

**Fig. 4:**
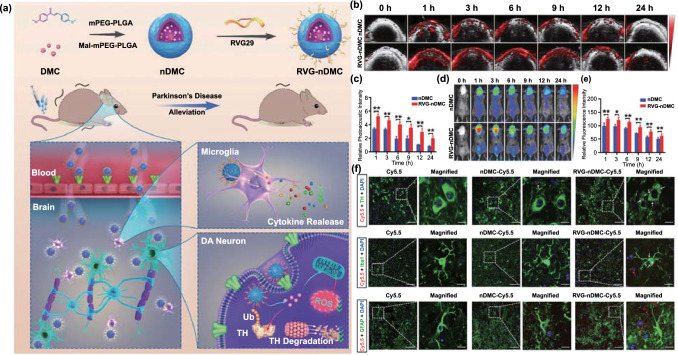
Example of RMT. **a** Preparation and proposed mechanism of RVG-nDMC for PD intervention. **b** Real-time photoacoustic imaging and **c** corresponding photoacoustic signal analysis of mice after intravenous injection of nDMC or RVG-Ndmc. **d** Real-time fluorescence imaging and corresponding fluorescence analysis of mice after intravenous injection of Cy5.5-labeled nDMC or RVG-nDMC. **f** Representative images of TH, Iba1, and GFAP staining in the SN of mice treated with Cy5.5, nDMC-Cy5.5, or RVG-nDMC-Cy5.5 at 6 h post-injection. White arrows in the enlarged parts of the right column show the presence of NPs in DA neurons and microglia. Red arrows show the presence of NPs outside astrocytes and microglia. Reproduced from Ref. [94]

Receptors on the surface of ECs are responsible for controlling the influx of macromolecules that maintain the homeostasis of the brain microenvironment [134]. Compared with the CMT strategy, the RMT strategy tends to provide better brain targeting, mainly owing to the relatively large number of receptors and the lack of concentration-dependent effects. RVG29, a new type of RMT-targeting peptide, can specifically recognize nicotinic acetylcholine receptors, which not only show high expression in ECs but also in astrocytes, microglia, and dopaminergic neurons. Therefore, nanoplatforms modified with RVG29 can achieve secondary targeting of PD lesions. In a recent study, Li et al*.* designed an RVG29-modified nanomedicine (RVG-nDMC) to deliver 4,4′-dimethoxychalcone (DMC) to immune cells and dopaminergic neurons in the brain in order to relieve neuroinflammation and oxidative stress in dopaminergic neurons (Fig. 4a) [94]. They first confirmed the brain targeting of RVG-nDMC through photoacoustic and fluorescent imaging. As shown in Fig. 4b-e, both imaging modalities showed a stronger signal intensity in the brain in the RVG-nDMC group than in the nDMC group, indicating that the BBB-crossing capability of this nanomedicine was due to RVG29. Subsequently, immunofluorescence analysis of brain slices also showed that RVG-nDMC displayed stronger targeting to dopaminergic neurons, microglia, and astrocytes (Fig. 4f). Behavioral experiments for evaluating the anti-PD effect of the nanomedicine showed that RVG-nDMC exerts a significant therapeutic effect on MPTP-induced PD symptoms. Through further mechanistic analysis, the group proved that RVG-nDMC reduces the levels of pro-inflammatory factors in the brain and also reduces mitochondrial dysfunction in the substantia nigra (SN), attenuating the loss of dopaminergic neurons.

According to previous reports, inflammation in the brain leads to a high expression of vascular cell adhesion molecule 1 (VCAM-1), intercellular adhesion molecule-1 (ICAM-1), and other inflammatory receptors [135–137]. Hence, the RMT strategy may effectively prevent the decrease in targeting efficiency caused by the expression of receptors in other tissues. In the study by Vladimir et al., tumor necrosis factor alpha (TNF-α) was used to induce brain inflammation, and subsequently, liposomes modified with different ligands were injected. They found that the accumulation of VCAM-1-modified liposomes in the brain was more than 300 times greater than that of immunoglobulin G-modified liposomes and also 27 times higher than that of transferrin/liposomes [138]. This ultrahigh delivery efficiency of the modified nanomedicine to the brain could be attributed to the highly specific expression of receptors in the area of inflammation and lack of specific recognition in other tissues. Evidence suggests that the neuroinflammation in PD can also induce a surge of inflammatory receptors [139]. Although no study has used these inflammatory receptors to achieve high-efficiency brain-targeted delivery of anti-PD drugs, this strategy is worthy of attention and warrants further research.

Overall, the RMT strategy offers higher brain-targeting efficiency and may also prevent the decrease in targeting efficiency caused by the non-specific expression of receptors. However, the RMT strategy also has some drawbacks, including the presence of a limited number of receptors and competition with endogenous substances. Further, the drug can be transported to lysosomes while crossing the BBB, leading to inactivation, with the drug failing to reach its target.

#### Nasal Delivery

In the early mid-nineteenth century, researchers found that dye injected into the subarachnoid space eventually appears in deep cervical lymph nodes, implying a direct connection between the nasal cavity and the brain. However, the potential of the nasal route for drug delivery to the brain was realized much later, and the method was first proposed in 1989 to treat CNS diseases [140]. Notably, drugs administered into the nasal cavity are easily eliminated by mucociliary hairs of the vestibular region. Therefore, a special nasal delivery device is required to ensure that the drug reaches the deeper respiratory and olfactory regions, from where it can move to the brain through the olfactory nerve pathway, trigeminal nerve pathway, lymphatic pathway, and cerebrospinal fluid. Importantly, during nasal delivery, a large proportion of the drug enters the brain via the neuronal route, and therefore, a nonsignificant part may also enter the systemic circulation [141].

**Fig. 5:**
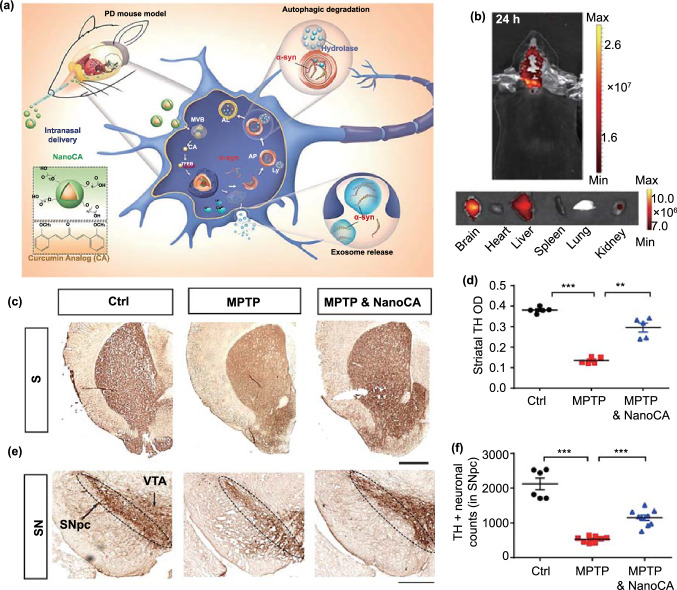
Example of nasal delivery. **a** Design of self-assembled NanoCA NPs for TFEB-regulated cellular clearance of *α*-syn in experimental models of PD. **b** Representative IVIS images showing the biodistribution of NanoCA@TPAAQ in mice after intranasal administration. **c** Representative photomicrographs of striatal TH immunostaining from the same animals. **d** Quantification of TH immunoreactivity in the striatum. TH immunoreactivity in midbrain sections from the same animals. **f** Quantification of surviving TH^+^ dopaminergic neurons in the SN. Adapted with permission from Ref. [109]. Copyright 2020 American Chemical Society

Today, nasal drug delivery has evolved into an important mode of treatment for several brain diseases, including glioblastoma [142], as well as neurodegenerative diseases [143]. Liu et al*.* synthesized a nanoparticle via the self-assembly of a curcumin analog (NanoCA) and attached PEG to its surface to improve its in vivo stability [109]. After reaching the target site, NanoCA degraded into free CA, promoting lysosomal elimination of α-syn by regulating helix-loop-helix transcription factor B (TFEB) (Fig. 5a). Moreover, NanoCA NPs were tagged with the fluorescent probe TPAAQ to assess its brain targeting. Twenty-four hours after nasal administration, NanoCA NPs were found to have efficiently targeted the brain (Fig. 5b). Next, the researchers used MPTP-induced models of PD to test the anti-PD activity of NanoCA. In the forced swimming test, the NanoCA-treated group behaved like the control group. In the open-field and gait dynamic tests, the NanoCA group performed only slightly worse than the control group but showed significantly better performance than the MPTP group. The immunohistochemical analysis of the SN also verified that the presence of NanoCA protected dopaminergic neurons, validating the anti-PD activity of NanoCA (Fig. 5c-f). However, the nasal mucosa decreases the bioavailability of common nanomedicines. Interestingly, thermosensitive hydrogels can effectively increase drug bioavailability after nasal delivery. In the nasal cavity, by controlling the phase transition temperature of the thermosensitive hydrogel within a suitable range, it can be easily transformed into a high-viscosity gel, extending the drug attachment duration. Further, an increase in nasal mucosal permeability increases drug bioavailability. However, for ease of preparation, it is usually necessary to control the phase transition temperature above 25 °C [144]. Therefore, the phase transition temperature of the most thermosensitive adhesives is within 25–37 °C. Rao et al*.* designed a ropinirole thermosensitive hydrogel with a phase transition temperature of 32.5 °C. They showed that nasal injection efficiently delivered the BCS class III drug to the CNS and improved both bioavailability and anti-PD efficacy [111].

Even though nasal delivery offers many advantages, it too has certain limitations such as device design restrictions for nasal delivery in humans. Moreover, there are currently some controversies related to the mechanisms of drug delivery through the nasal route. Intracellular mechanisms suggest that post internalization in olfactory sensory neurons, the drug is transported along the axon to the synapse, where it is released into the olfactory bulb, allowing final delivery to the brain. In contrast, the paracellular mechanisms indicate that the drug first enters the paracellular space and is then delivered to the lamina propria of the nasal epithelium. From there, the drug enters the subarachnoid space [140]. Therefore, the further elucidation of the anatomical mechanism is vital for the rapid clinical transformation of nasal drug delivery. In addition, gels with appropriate viscosity are more suitable for nasal delivery than liquid formulations because they reduce drug loss and facilitate sustained drug release.

#### Biomimetic Drug Delivery

The concept of biomimetic drug delivery was proposed by Zhang et al*.* in 2011 [145], who showed that NPs coated with red blood cell membranes exhibit significantly increased retention times in vivo. This discovery undoubtedly opened a new avenue for the development of nanomedicines. Encapsulating NPs with endogenous biological membranes confer unique advantages such as immune escape and prolonged circulation durations. Further, the membranes add physiological functions to the nanomedicine; for instance, platelet and neutrophil membranes, owing to their inflammatory tendencies, function as natural anchors for tumor cells [146–148]. Moreover, even cell membranes that lack specialized targeting abilities can be surface modified to enable specific delivery.

Currently, biomimetic drugs are receiving great attention for their capability to cross the BBB. For instance, Zou et al*.* used a targeting ligand, angiopep-2, to enable RBC membrane-coated NPs to cross the BBB [149]. In a similar study, Fu et al*.* used T7 and NGR short peptides to modify the surface of RBCs [150]. Notably, the brain metastasis of breast cancer, lung cancer, and other tumors indicates that these cancer cells can easily traverse the BBB. Therefore, the cell membrane of these cancer cells can be used as camouflage to help NPs traverse the BBB. However, so far, there have been no relevant reports of this method being used for PD treatment.

Exosomes are cell-excreted stable nanoscale lipid vesicles (30–200 nm diameter) that can be used as biomimetic nanoplatforms [125]. Exosomes have the characteristic protein content of their parent cells, easing their transport across the BBB. Likewise, they can improve the targeting of nanomedicines. Recent studies have reported exosome-mediated drug delivery to the brain; however, the exosomes used in these studies were surface modified with ligands to help them traverse the BBB. Perets et al*.* used mesenchymal stem cell (MSC)-secreted exosomes to coat gold NPs (GNPs), and using brain imaging, showed that the MSC-exo-GNPs could easily aggregate in the brain in models of various degenerative diseases, including PD [151]. Likewise, intravenously injected blood exosomes loaded with dopamine were found to be effective against 6-OHDA-induced PD [116]. In another study, Ren et al*.* showed that RVG-attached HEK293T cell exosomes enhanced delivery efficiency to PD lesions [115]. Although all these studies provide a reference for the treatment of PD using exosomes, the strategy of linking ligands for BBB targeting has shortcomings similar to those of the RMT strategy, which need to be addressed in the future.

**Fig. 6:**
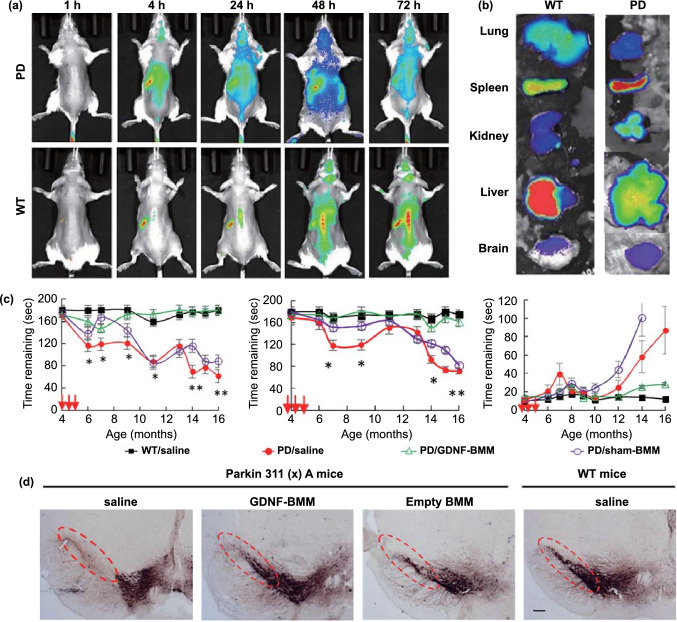
Biomimetic-delivery strategy for BBB crossing. **a** Parkin Q311(X)A mice (4 Mo. of age) were *i.v.* injected with 6 × 10^6^ DIR-macrophages and imaged using IVIS. **b** At the end point (72 h), mice were sacrificed, and perfused; the main organs were removed, and images were obtained using IVIS. **c** Behavioral tests demonstrating the preservation of locomotory function in Parkin Q311(X) mice upon treatment with GDNF-macrophages at an early stage of disease. **d** GDNF-BMM could protect dopaminergic neurons in Parkin Q311(X)A mice. Adapted from Ref. [156]

In addition, due to neuroinflammation, immune cells can permeate into the parenchyma in patients with PD. This feature can also be used to improve brain delivery. Current PD treatment studies based on this strategy typically use macrophages as drug carriers [152–155]. In a recent study, macrophages loaded with glial cell line-derived neurotrophic factor (GDNF) were used for the treatment of PD [156]. 1,1'-dioctadecyltetramethylindotricarbocyanine iodide (DiR)-labeled macrophages were used to examine the BBB permeability. The results showed that the fluorescence levels in the brains of PD mice were obviously higher than those in WT mice (Fig. 6a-b), indicating the macrophages successfully crossed the BBB. Behavioral parameters were found to be significantly improved in Parkin Q311(X)A mice after treatment with GDNF-loaded macrophages (Fig. 6c). Immunohistochemical results showed that the number of TH^+^ neurons in the substantia nigra increased in the GDNF-macrophage group (Fig. 6d). The excellent curative effect was not only attributed to the therapeutic effect of GDNF, but also to the anti-inflammatory effect of macrophages themselves. In addition to macrophages, microglia and neutrophils are also known to cross the BBB. Among them, microglia have shown potential in PD treatment [157]. However, neutrophils have only been studied from the perspective of improving the surgical prognosis of glioma [158]. Importantly, because lysosomes tend to degrade drugs, during cell-based drug delivery, the intracellular inactivation of drugs needs to be prevented. Unfortunately, the existing studies have so far not considered this caveat. Nevertheless, the pretreatment of cells with lysosome inhibitors could help in achieving this defect.

Biomimetic drugs are novel drugs that have emerged in the past decade. They provide an excellent delivery platform for the treatment of brain diseases. However, so far, there have been few reports on the use of such drugs with BBB-crossing capability for the treatment of PD. In addition to reports on cell-based drug delivery, recent reports also suggest that different biofilms can be fused to form a composite membrane that possesses the physiological functions of the parent membranes [159]. This new approach may allow secondary targeting in PD. For example, membranes of tumor cells that can traverse the BBB can be fused with platelet membranes to produce a biomimetic drug that targets neuroinflammation.

### Physical Strategies

Over recent years, multiple physical strategies have been found to improve the permeability of the BBB. Although most of these strategies are in infant stage, safety evaluations have shown that these strategies have excellent biocompatibility and great potential for clinical application. In this section, we will review these strategies.

#### Magnetic Targeted Drug Delivery

Iron can be easily utilized to generate iron oxide NPs (IONPs). Furthermore, after biodegradation, these IONPs release iron, which is considered safe by the American Food and Drug Administration. Therefore, the use of IONPs is widely accepted in the biomedical field. Moreover, given their intrinsic magnetic properties, IONPs are also often used in MRI. More importantly, IONPs can be embedded in silica or organic polymers to generate larger NPs with a better drug loading capacity. Fascinatingly, the application of an external magnetic field disrupts the TJs in the BBB, allowing drug-containing IONPs to cross the barrier [160, 161].

**Fig. 7:**
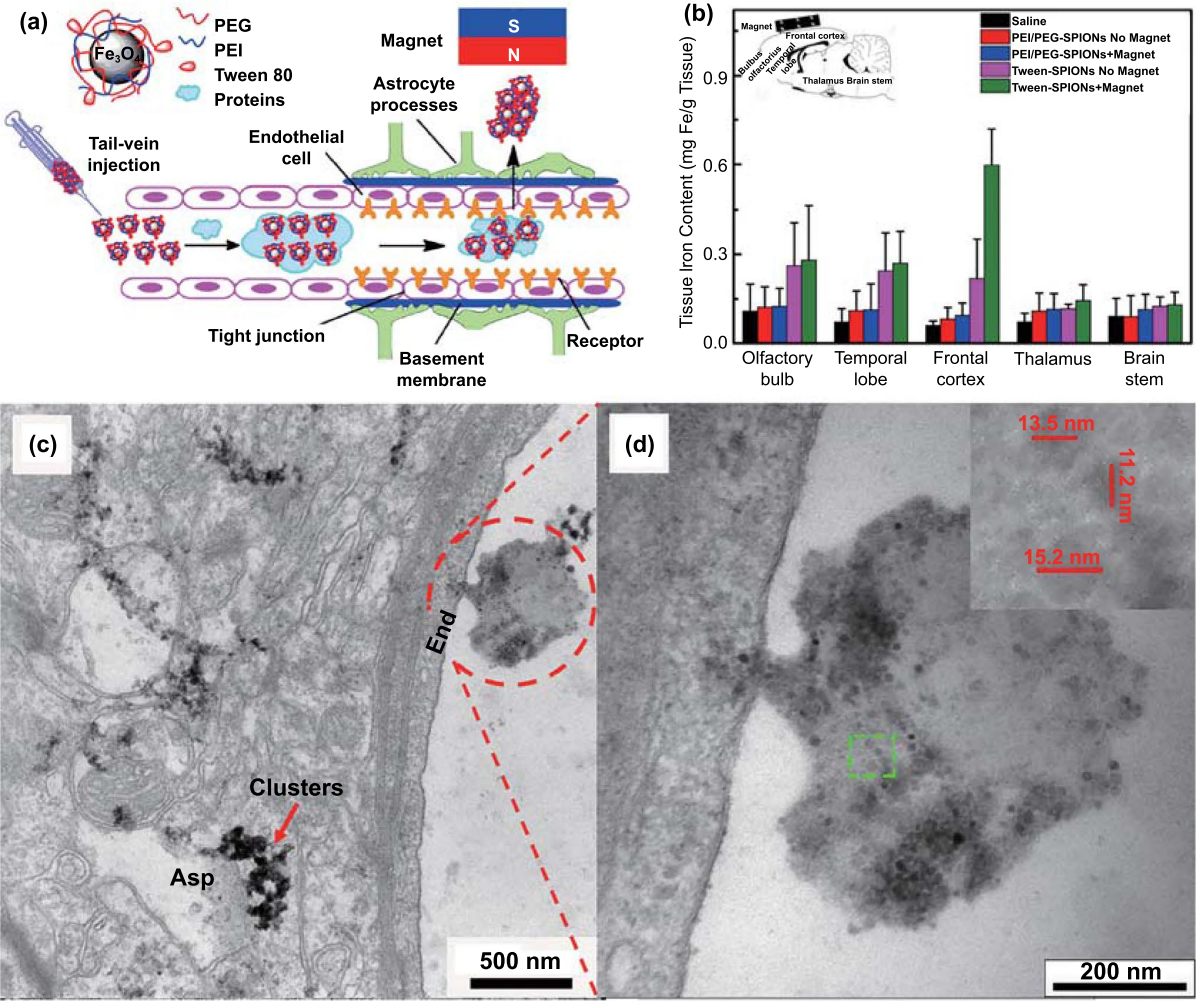
Example of magnetic force-mediated brain delivery. **a** Schematic illustration for Tween-SPIONs crossing the BBB in the presence of a magnet. **b** The distribution of iron in different brain areas in rats 2 h after tail vein injection. The inset image indicates the position of the magnet. **c** SPIONs enter the brain by crossing the BBB. Asp: astrocyte processes, End: endothelial cell. **d** Higher magnification image; the inset shows the size of the SPIONs. Adapted with permission from Ref. [162]. Copyright 2016 American Chemical Society

For example, Tan et al*.* wrapped IONPs in PEG and polyethyleneimine (PEI) and intravenously injected these composite IONPs into mouse models before providing an external magnetic field to the brain (Fig. 7a) [162]. To prove that the external magnetic field is helpful for brain delivery, the iron levels in different brain regions were detected after 2 h. The results showed that the iron content in the brain of the mice was significantly higher in the magnetic field treatment group than in non-magnetic field group, indicating that the external magnetic field successfully increased the accumulation of IONPs in the brain (Fig. 7b). In addition, electron microscopy analysis indicated that the composite IONPs first formed a protein crown-nanocomplex during systemic circulation, and then, under the action of an external magnetic field, they successfully penetrated vascular endothelial cells and entered the brain parenchyma (Fig. 7c). However, it must be noted that due to the formation of these nano-protein crown complexes, IONPs are more easily phagocytosed by macrophages and thus have a shorter half-life in vivo. Therefore, various methods, such as modification with organic polymer materials and biofilm camouflage, are adopted to increase their half-life. Recently, Kim et al*.* successfully combined IONPs with biomimetic drugs to prepare a nanomedicine that easily traverses the BBB under the application of an external magnetic field [163]. First, IONPs were added to MSCs to produce therapeutic biomolecules, and after a period of incubation, the cell membranes of MSCs were destroyed to isolate nuclei via centrifugation. Subsequently, the cell membrane enclosing the therapeutic biomolecules and IONPs was compressed by a filter membrane to generate magnetic nanovesicles (MNVs). These MNVs retained the magnetism-mediated targeting capability of IONPs and also had the systemic circulation characteristics of biomimetic drugs. To investigate the brain-targeting capability of MNVs under the application of an external magnetic field, MNVs were labeled with the fluorescent lipophilic dye VivoTrack 680. Notably, the researchers used a special 3D-printed helmet to produce a magnetic field around the left side of the brain in mice. After intravenous injection, the special helmet promoted the accumulation of MNVs in the left side of the brain, indicating that the external magnetic field enhanced the BBB-crossing activity of IONPs. Notably, this strategy has also been used in PD theranostics. Gao et al*.* prepared resveratrol-loaded liposomes, which were then wrapped with chitosan to further load Fe_3_O_4_ NPs (Res-lips@Fe_3_O_4_) [119]. In this drug delivery system, Fe_3_O_4_ NPs improved both magnetic force-induced BBB permeability and MRI diagnosis in PD patients. In MRI imaging, parameters such as fractional anisotropy (FA) values and T2 relaxation time are used for diagnosis. The FA value in the region-of-interest is lower in the brains of PD patients than in healthy brains. The T2 relaxation time is also longer in PD patients owing to an increase in the interstitial water content in the lesion area. In a previous study, it was found that compared with the PD model group and the Res-lips@Fe_3_O_4_ only group, the Res-lips@Fe_3_O_4_ combined with external magnetic field application group exhibited higher FA values and lower T2 relaxation times. This was consistent with the results from the sham group, which also demonstrated the improved anti-PD efficacy of Res-lips@Fe_3_O_4_. Overall, the findings from this study strongly advocate for magnetic force-dependent brain-targeted drug delivery.

The feasibility and effectiveness of magnetic force-dependent IONP-based brain-targeted drug delivery have been validated by many studies. Early diagnosis is one of the major challenges in PD, and IONPs can easily be used to integrate both PD diagnosis and treatment. However, the brain delivery of IONPs can also be affected by non-specific external magnetic fields. In animal studies, a special helmet was used to make the targeting of specific brain lesions easier because the exact location of the lesion was well known. The development of case-specific instruments for treating human PD patients is not feasible. On the other hand, the metabolites of IONPs include multivalent iron. Excess iron could promote the Fenton reaction in PD lesions, that is, lead to the production of more ROS, which is unfavorable for the treatment of PD. Therefore, IONP-mediated brain-targeted delivery should be optimized further.

#### Photothermal Brain Delivery

Phototherapy, including photothermal therapy (PTT), is a spatiotemporally controlled method for tumor treatment. In PTT, a photosensitizer is first injected into the body, and then, a specific wavelength of near-infrared (NIR) light is focused on the target tissue. The photosensitizer absorbs light energy and converts it into energetic electrons or holes through localized surface plasmon resonance, or it produces energy via electron transition and transfers energy to its surroundings through vibration energy scattered by the crystal lattice; thereby, the photosensitizer causes an increase in temperature [164, 165]. Given its potential and high clinical value, this strategy is under validation in several clinical trials [166].

Recently, Chen et al*.* demonstrated a novel application of the photothermal effect [31]. After intravenously injecting black phosphorus nanosheets (BPNSs, a type of nanomaterial with a light-to-heat conversion effect), they irradiated the brains of mice with 808 nm NIR in order to maintain a temperature of 41–43 °C. Then, they injected Evans blue dye intravenously. They found significant Evans blue accumulation in the brains of mice treated with BPNSs + NIR. This important study proved that the photothermal effect could increase the permeability of the BBB and could therefore be a new strategy for brain drug delivery. Moreover, this strategy is precise and BBB permeability can easily be controlled by adjusting the intensity and duration of NIR irradiation. Based on this theory, Zhou et al*.* used Ru NPs (RuNP) with excellent photothermal conversion efficiency as drug carriers for the targeted delivery of nerve growth factors in an Alzheimer’s disease model [167]. To prove that the increase in local temperature can indeed enhance BBB permeability, they used an infrared camera and recorded the changes in brain temperature after the intravenous injection of RuNP followed by 5-min NIR irradiation to maintain the temperature at 41–43 °C (as earlier). Subsequently, they found consistent results in both the Evans blue test and in vivo fluorescence imaging. The intravenous injection of RuNP into mice improved BBB permeability only in the presence of NIR irradiation. Additionally, they also confirmed the safety of this strategy with respect to brain damage or thrombosis. However, one important aspect they ignored was the loss of BBB selectivity owing to the overall increase in permeability, even for toxic substances.

**Fig. 8:**
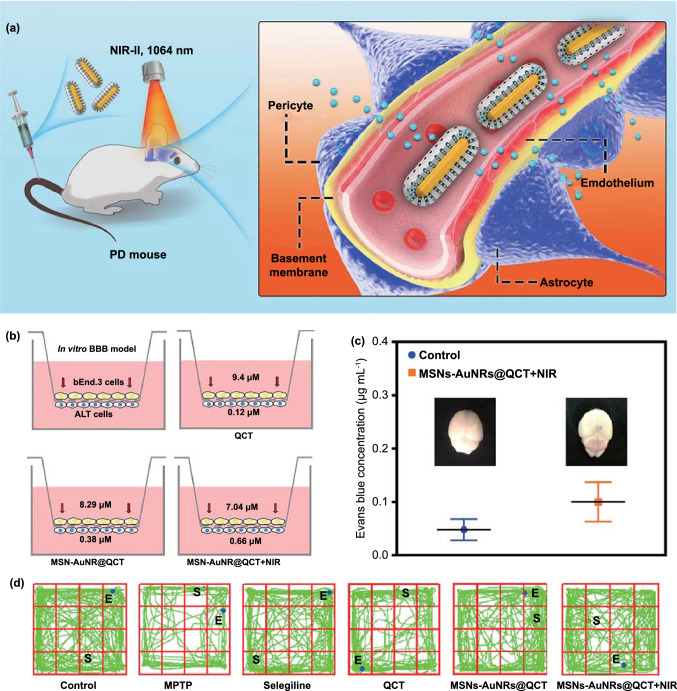
Example of photothermal brain delivery. **a** The proposed mechanism underlying MSNs-AuNRs@QCT penetration across the BBB under NIR-II (1064 nm) laser irradiation. **b** Overview of the in vitro BBB Transwell system used to gauge the penetrative capabilities of MSNs-AuNRs@QCT, and the final concentration of QCT in the apical and basolateral chambers in the BBB transwell system. **c** Photothermal effect on BBB permeability after MSNs-AuNRs@QCT injection and 1064 nm laser irradiation, examined using Evans blue as a BBB permeability indicator. **d** Representative movement of mice in an open-field box (green). Adapted with permission from Ref. [121]. Copyright 2020 American Chemical Society

Importantly, demonstrating the safety of this strategy, Xiong et al*.* showed that the photothermal effect-mediated increase in BBB permeability is reversible, and BBB permeability returns to original levels 48 h after treatment [120]. Moreover, they adopted this strategy for PD treatment, using an auxiliary brain nanodelivery system based on BPNSs. The BPNSs were modified with lactoferrin (Lf) and loaded with paeoniflorin (Pae) to prepare Lf-BP-Pae NPs. First, they tested whether BPNSs or Lf-BPNSs increase the temperature of the mouse brain. Pae was replaced with the fluorescent dye Cy5, and it was found that different treatments led to distinct levels of fluorescence intensity in the brain, with Lf-BP-Cy5 + NIR providing the highest intensity. Finally, the anti-PD effect of the Lf-BP-Pae + NIR strategy was tested. Based on immunofluorescence detection of TH^+^ neurons in the SN, they found that Lf-BP-Pae + NIR provided a significantly better PD-alleviating effect than did treatment with Lf-BP-Pae alone. Although these reports prove the safety and feasibility of the photothermal strategy, this method has the same limitations as tumor PTT. Moreover, the commonly used light is NIR-I (700–1000 nm), which has limited penetration and may not reach an optimum depth. In contrast, NIR-II illumination provides stronger tissue penetration and is more suitable for inducing the photothermal effect in vivo. Liu et al*.* used mesoporous silica (MSNs) to encapsulate gold nanorods (AuNRs) with a photothermal effect and loaded them with quercetin (QCT). The resulting MSNs-AuNRs@QCT particles were used to treat an MPTP-induced mouse model of PD (Fig. 8a) [121]. Then, they used 1064 nm NIR-II to induce AuNR-mediated photothermal effects, and they observed a successful increase in the efficient delivery of QCT and BBB permeability both in vitro and in *vivo* (Fig. 8b-c). Therefore, in the open-field experiment, the MSNs-AuNRs@QCT group receiving NIR showed the most similar trajectory relative to the control group, indicating it had the best curative effect (Fig. 8d).

In summary, the photothermal strategy has been proved to enhance BBB permeability, but the mechanism underlying its effects remains unknown. More attention should be paid to select suitable illumination conditions, as crossing the time and intensity thresholds of NIR irradiation may have unpredictable adverse effects, such as irreversible BBB damage and thrombosis.

#### Focused Ultrasound-Mediated Brain Delivery

Ultrasound can generate a variety of bioeffects via thermal and mechanical energy and is therefore widely used in the medical field. Previously, researchers found that ultrasound can interfere with the integrity of the BBB, increasing its permeability, but that such effects are often accompanied by brain damage [168]. In 2001, Kullervo et al*.* used microbubbles (MBs, balls of gas wrapped in a semi-rigid lipid or albumin shell, 1–10 μm in size) and demonstrated for the first time that focused ultrasound (FUS) can effectively increase BBB permeability without causing any brain damage [169]. In another important study, Zhang et al*.* observed that the Evans blue dye failed to enter the brain parenchyma after 2 h of FUS + MBs treatment, indicating that the effect of FUS is reversible [170]. Moreover, MBs have excellent biocompatibility and safety.

**Fig. 9:**
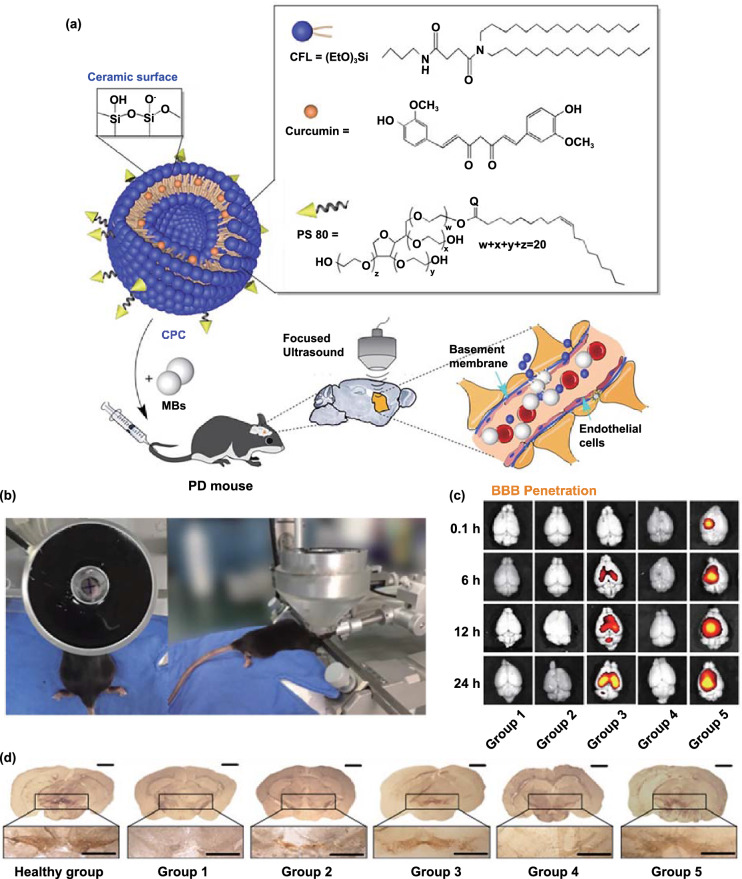
Example of focused ultrasound-mediated brain delivery for PD treatment. **a** Improvements in the therapeutic efficacy of curcumin in PD mouse models obtained using CPC combined with ultrasound-targeted microbubble destruction. Schematic of the chemical composition of CPC and the non-invasive localized delivery of CPC NPs to the mouse brain via the ultrasound-targeted microbubble destruction technique for PD therapy. **b** Photograph of the ultrasound-targeted microbubble destruction setup for the local treatment of the corpus striatum in C57BL/6 mice. **c** Representative ex vivo fluorescence images obtained at 0.1, 6, 12, and 24 h after intravenous administration (*n* = 6 per group at each time point). **d** Representative immunohistochemical staining images of TH^+^ neurons in the SN in mouse brain sections from different groups (*n* = 6 per group). Group 1: control; Group 2: only curcumin-loaded cerasomes with no PS 80; Group 3: only CPC with 5% PS 80; Group 4: 5% PS 80-modified cerasomes with no curcumin in combination with ultrasound-targeted microbubble destruction; Group 5: CPC with 5% PS 80 in combination with ultrasound-targeted microbubble destruction. Adapted with permission from Ref. [125]

In recent years, the MBs + FUS strategy has been widely used in the treatment of various CNS diseases [171–173]. Using this strategy, Niu et al*.* achieved high-efficiency brain-targeted delivery of fibroblast growth factor-20-loaded liposomes and enhanced the in vivo anti-PD efficacy [123]. Similar to magnetic force-mediated BBB crossing, confocal ultrasound (CFU) is also a precise technique. Zhang et al*.* used curcumin-loaded cerasomes (CPCs) along with polysorbate 80-modified MBs and used FUS (PS 80) to achieve improved anti-PD effects (Fig. 9a) [125]. To verify the precision of the MBs + FUS strategy, they applied CFU to the left striatum and then imaged mouse brains ex vivo (Fig. 9b. The results showed that FUS significantly increased the accumulation of curcumin in the left side of the brain (Fig. 9c). As expected, the combination of FUS and MBs significantly increased the permeability of the BBB. Moreover, immunohistochemistry of tissue from the SN showed that MBs + FUS-mediated delivery of PS 80-CPC-curcumin greatly increased the number of TH^+^ neurons (Fig. 9d).

There have been several preliminary clinical attempts to temporarily open the BBB using this new strategy, with no reported patient discomfort [174]. FUS combined with MBs has been reported to be safe and effective for PD patients, with the BBB recovering 24 h after treatment [175]. However, it is necessary to ensure the complete safety of this technique before wide clinical application. Particularly, the optimal range of ultrasound frequency needs to be confirmed. If the frequency used is too high, it may result in major safety concerns, whereas if it is too low, the BBB permeability would not increase. In addition, apart from its use an adjuvant drug delivery system in PD, FUS is effective in reducing dyskinesia and therefore can reduce PD symptoms via theranostics [176, 177].

### Others

In addition to the strategies mentioned above, there are certain less commonly applied and researched strategies for drug delivery to the brain. These can be divided into three categories. The first category involves the use of a self-propelled nanocarrier that can directly enter various organs through blood vessels without being affected by blood flow and local tissue structure. Joseph et al*.* designed a nanocarrier that is self-propelled in the presence of a glucose gradient and could be used for gene delivery to the CNS [178]. The second category involves the injection of mannitol or arabinose into the body to increase the osmotic pressure of brain blood vessels, leading to the contraction of ECs [179, 180]. This contraction results in the down-regulation of TJs and an increase in BBB permeability. Moreover, mannitol inhibits the aggregation of α-syn protein and has anti-PD activity [181]. The third and final category is the use of the ear and CSF connection to bypass the BBB. The ear has also become a potential injection route for PD treatment [182]. Though most of these strategies are in the initial stages of research and have not been applied for the clinical treatment of PD, they can be developed further to devise novel strategies.

## Nano-Biological Effects of Nanomaterials Used for PD Treatment

Conventionally, functionalization has been the primary tool for developing novel nanomaterials. However, interactions between the nanomaterial and the internal environment of the body, including interactions with proteins and redox reactions, did not receive much attention, even though they can be exploited for theranostics.

The upregulation and aggregation of α-syn are the main characteristics of PD. Therefore, patients with PD show enhanced intercellular transfer of α-syn, resulting in the continuous activation of microglia. Such microglial activation promotes the apoptosis of dopaminergic neurons via an increase in the production of neurotoxic substances such as ROS. To counter this problem, several advanced treatment strategies involving the removal of excess ROS, inhibition of α-syn aggregation, and regeneration of neurons have been developed. In this section, we discuss the biological effects of nanomaterials that can be used in such advanced treatment strategies. A list of these materials is presented in Table 3.

**Table 3:** Nanomaterials with anti-Parkinsonian nano-bio effects

Nanoplatform	Size (nm)	Zeta potential (mV)	PD model	Nano-biological effects	Mechanism	References
Mn_3_O_4_ nanoparticle	180	N/A	MPP^+^	Clearance of excessive ROS	Mimic superoxide dismutase (SOD), catalase (CAT), and glutathione peroxidase (GPx) by switching between Mn^2+^ and Mn^3+^	[183]
Ceria nanoparticle	400	− 20	MPTP	Clearance of excessive ROS	Mimic SOD and CAT by switching between Ce^3+^ and Ce^4+^	[184]
Graphene oxide quantum dot	Lateral size 20–40, thickness 4.18–5.19	N/A	MPP^+^	Clearance of excessive ROS	Mimic CAT	[185]
Cerium oxide nanoparticle	25	− 12.9	6-OHDA	Clearance of excessive ROS	Mimic SOD and CAT by switching between Ce^3+^ and Ce^4+^	[186]
Carboxyfullerene	N/A	N/A	MPTP	Clearance of excessive ROS	Clear ROS	[32]
PtCu nanoalloys	32.1 ± 4.5	− 15.3 ± 1.5	PFFs	Clearance of excessive ROS	Mimic POD, CAT, and SOD	[187]
Cu_x_O nanoparticle cluster	65 ± 7	N/A	MPTP	Clearance of excessive ROS	Mimic SOD, CAT, GPx and peroxidase (POD) by switching between Cu^2+^ and Cu^+^	[33]
Yb^3+^, Er^3+^ codoped cerium oxide upconversion nanoparticle	18.6 ± 0.2	N/A	MPTP	Clearance of excessive ROS	Mimic CAT and SOD	[188]
2D V_2_C Mxenzyme	N/A	N/A	MPTP	Clearance of excessive ROS	Mimic SOD, CAT, POD, and GPx	[189]
Graphene quantum dot	Lateral size 1.74 and thickness 2	N/A	PFFs	Inhibition of α-syn aggregation	Interaction with α-synuclein monomers via electrostatic force and dissociation of α-synuclein fibrils via the hydrophobic effect	[190]
Gold nanocluster	2.5 ± 1.0	N/A	MPTP	Inhibition of α-syn aggregation	N/A	[191]
CdTe/CdS/ZnS quantum dot	N/A	N/A	MPP^+^	Inhibition of α-syn aggregation	Active autophagy to clear α-synuclein	[192]
Superparamagnetic iron oxide nanoparticles	N/A	− 30	N/A	Inhibition of α-syn aggregation	Interaction with α-synuclein monomers via electrostatic force	[193]
Lys-coated Fe_3_O_4_ nanoparticle	5–7	+ 26	N/A	Inhibition of α-syn aggregation	Interaction with α-synuclein monomers via electrostatic force	[194]
Gold nanoparticle	35.6 ± 8.8	+ 16.2 ± 5.2	MPTP	Dopaminergic neuron regeneration	Enhance neuronal gene expression of somatic fibroblasts under electromagnetic force application	[195]
Nanomatrix based on silica	N/A	N/A	6-OHDA	Dopaminergic neuron regeneration	Activate the integrin β1-RhoA-GLI1 signaling pathway in neural stem cells	[196]
Dextran-coated iron oxide nanoparticle	N/A	N/A	6-OHDA	Dopaminergic neuron regeneration	Enhance the migration of hMSCs toward damaged DA neurons and transdifferentiate hMSCs into DA-like neurons	[197]

### Clearance of Excessive ROS

**Fig. 10:**
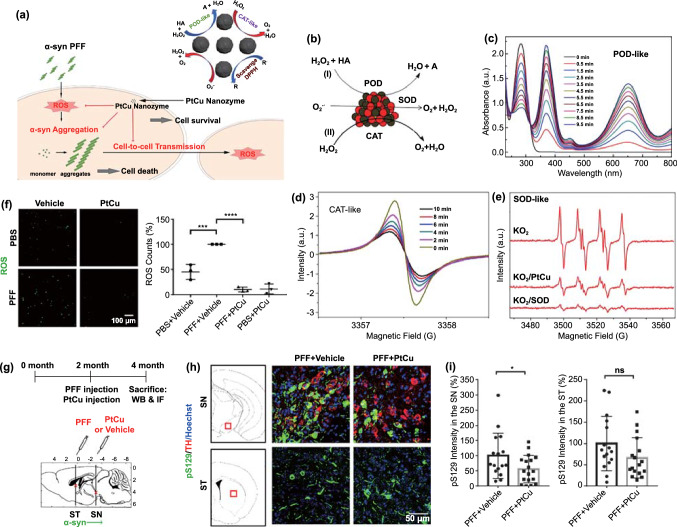
Example of nano-bio effects resulting in the clearance of excessive ROS. **a** Schematic showing how the PtCu nanozyme scavenges ROS and prevents α-synuclein-induced pathology, neurotoxicity, and cell-to-cell transmission in vitro and in vivo. **b** Schematic showing how PtCu Nas mimic three redox enzymes (POD: peroxidase, SOD: superoxide dismutase, CAT: catalase). **c** UV–Vis spectra of TMB in the presence of H_2_O_2_ catalyzed by POD-like PtCu Nas. **d** CAT-like activity of PtCu NPs in reducing H_2_O_2_, demonstrated by electron spin resonance (ESR) oximetry; the evolution of the ESR spectra of PDT over time in the presence of 2 Mm H_2_O_2_ before and after the addition of PtCu NPs in a closed chamber can be observed. **e** The SOD-like activity of PtCu Nas in reducing superoxide levels, demonstrated using ESR spectroscopy. **f** PtCu Nas reduce PFF-induced ROS and quantifies ROS levels. **g** Timeline of PFF animal experiments with PtCu Nas treatment (top) and the stereotaxic injection sites for PFF and PtCu/Vehicle (bottom). Two-month-old mice were stereotaxically injected with PFF and PtCu/Vehicle and were sacrificed after two months. **h** Ps129 immunostaining in the substantia nigra (SN) and striatum (ST). Brain sections were stained with anti-Ps129 and anti-TH (tyrosine hydroxylase) antibodies. **i** Quantification of Ps129 immunostaining. Adapted with permission from Ref. [187]. Copyright 2020 Elsevier Ltd. All rights reserved

Singlet oxygen, superoxide anions, hydrogen peroxide, and hydroxyl free radicals are the major ROS. Under normal physiological conditions, these species get eliminated by endogenous biological antioxidants, such as superoxide dismutase (SOD), glutathione peroxidase (GPx), catalase (CAT), and peroxidase (POD). However, in PD, there is a ROS imbalance in the brain, and excessive ROS induces oxygen stress, damaging various biomolecules such as DNA, proteins, and lipids and eventually causing neuronal cell death [198, 199]. An artificial increase in oxidoreductases can be used to eliminate excessive ROS in PD. Natural antioxidants, such as vitamin C, cannot reach the lesion area because of the BBB. The use of a nanoplatform can help in solving this problem. As for drug delivery, molecules that can directly or indirectly scavenge ROS can be loaded onto nanoplatforms modified with targeting ligands, such as PECAM and ICAM, to efficiently deliver them to inflammation sites and reduce ROS levels [135, 136, 200, 201]. However, it is difficult to completely encapsulate all oxidoreductases into nanoplatforms, which reduces potency. At present, the main method to address this problem is the use of metal materials that are prone to valence conversion during degradation for the direct preparation of NPs with BBB-crossing capabilities. For example, Kwon et al*.* used ceria NPs that mimicked SOD function and successfully attenuated MPTP-induced dopaminergic neuron damage upon a valency change [184]. Furthermore, owing to its superior oxidation resistance, magnesium simulates SOD, CAT, and GPx and therefore has great anti-PD potential [183]. However, uncontrolled active valence transitions in metal NPs can lead to unpredictable biological behavior. To solve this, Liu et al*.* proposed that metals with stable zero valence and metal ions with adjustable catalytic activity should be used in combination with each other. They prepared antioxidant PtCu bimetallic nanoalloys (PtCu Nas) to alleviate the ROS-induced neuronal loss in an α-syn preformed fibrils (PFFs) model (Fig. 10a). Moreover, in vitro findings suggested that these PtCu Nas behaved like POD, CAT, and SOD, thereby effectively reducing the levels of hydrogen peroxide and superoxide anions (Fig. 10b-e). Furthermore, in vivo experiments revealed that PtCu Nas successfully eliminated excessive intracellular ROS and reduced cytotoxicity (Fig. 10f). In the next series of experiments, PFFs and PtCu Nas were injected into the brains of mice, and two months later, the brains were isolated for immunofluorescence analysis (Fig. 10g). The researchers found that PtCu Nas effectively reduced the α-syn levels in the SN and increased the number of TH^+^ neurons, indicating that they had a strong anti-PD nano-biological effect (Fig. 10h-i).

Likewise, some non-metal nanomaterials also possess similar abilities. One such material is graphene oxide quantum dots (GOQDs), which are a classic graphene-based nanomaterial with redox capability. Using a zebrafish model, Ren et al*.* showed that GOQDs can degrade H_2_O_2_ into H_2_O and O_2_ and thereby alleviate MPTP-induced neurotoxicity without causing any adverse effects [185]. However, in another study by the same group, graphene oxide nanosheets were found to cause oxidative stress and neurotoxicity in zebrafish brains [202]. These inconsistencies can be attributed to the differences in the absorption efficiency and degradation capabilities of different cell types and the sizes of the nanomaterials, even though the same type of nanomaterial with the same degradation characteristics was used.

The other non-metal nanomaterials that can scavenge ROS include the carbon-based nanomaterial carboxyfullerene and black phosphorus, which have received widespread attention in recent years [31, 32]. These nanomaterials have certain antioxidant features. They can undergo reduction reactions, and their crystal structure facilitates the speedy transfer of electrons. Moreover, these non-metal nanomaterials have excellent biocompatibility and their degradation products, which are mostly buffer salts, are much safer. These properties further emphasize the potential of their clinical application.

Despite their excellent antioxidant properties, there are certain safety concerns over the use of nanomaterials. Recent studies have highlighted that nanomaterials, especially metal nanomaterials, can induce oxidative stress and inflammatory responses and alter the expression of neurotransmitters [203]. Notably, metal ion homeostasis is critical for normal brain function. An increase in the Fenton reaction (due to increased Cu^2+^ and Cu^+^ content) can lead to the generation of excessive ROS. Therefore, to obtain high anti-PD efficacy, it is necessary to cautiously regulate the impact of nanomaterials and their degradation products on the homeostasis of the brain microenvironment.

### Inhibition of α-Synuclein Aggregation

The α-syn protein was first identified and described by Maroteaux et al*.* in 1988. α-syn is a soluble protein that is abundant in the brain, and it regulates neuroplasticity and dopamine neurotransmitters [204]. The protein is composed of 140 amino acids and has an amphipathic N-terminal and central hydrophobic region. Importantly, it is strongly acidic at the C-terminal. Under normal physiological conditions, the transient interaction between the C-terminal and the N-terminal stabilizes the protein [205]. However, under conditions of overexpression, gene mutation, and Serine 129 phosphorylation, α-syn aggregates abnormally and eventually forms neurotoxic oligomers, fibrils, and Lewy bodies [206, 207]. Therefore, the removal of excessive α-syn monomers or decomposition of its fibrils can be effective against PD.

**Fig. 11:**
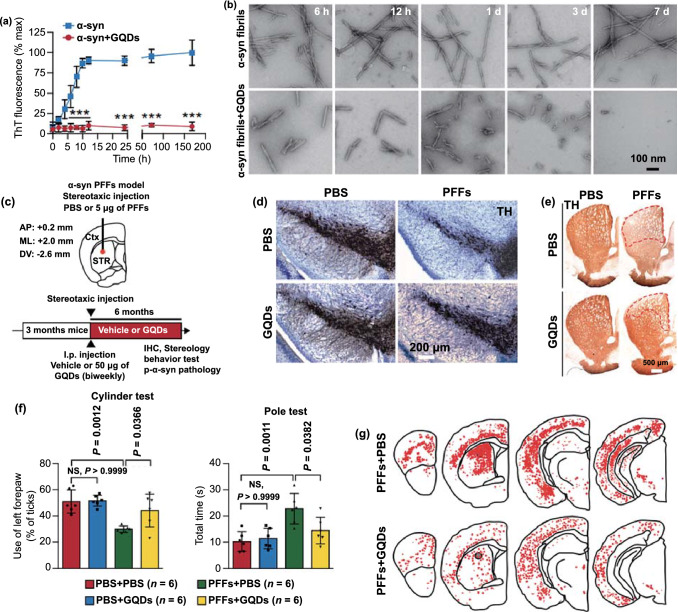
Example of nano-bio effects resulting in the inhibition of α-syn. **a** Kinetics of α-syn fibrillization monitored using a ThT fluorescence assay. **b** TEM images of preformed α-syn fibrils at various time points (6 and 12 h and 1, 3, and 7 days) in the absence (top) and presence (bottom) of GQDs. **c** Schematic illustration of stereotaxic intrastriatal injection coordinates for α-syn PFFs (5 μg) in C57BL/6 mice. As a treatment, 50 μg of GQDs or PBS were i.p. injected biweekly for 6 months. AP, anteroposterior; ML, mediolateral; DV, dorsoventral; Ctx, cortex; STR, striatum; IHC, immunohistochemistry. **d** Representative TH immunohistochemistry images of the SN from the α-syn PFF-injected hemisphere in the absence (top) and presence (bottom) of GQDs. **e** Representative TH immunohistochemistry images of the striatum from the α-syn PFF-injected hemisphere. **f** Assessments of behavioral deficits based on forepaw activity in the cylinder test (left) and the ability to grasp and descend from a pole (right). **g** Distribution of LB/LN-like pathology in the CNS of α-syn PFF-injected mice (p-α-syn positive neurons, red dots; p-α-syn positive neurites, red lines). Adapted with permission from Ref. [190]. Copyright 2018 Nature Publishing Group

Recent reports suggest that certain nanomaterials can react with α-syn and thereby alleviate the symptoms of PD. For example, Gao et al*.* showed that gold nanoclusters significantly reverse dopaminergic neuron loss and relieve PD symptoms by effectively inhibiting the aggregation and fibrosis of α-syn [191]; however, the exact mechanism remains unknown. In another important study, Kim et al*.* showed that graphene quantum dots (GQDs) inhibit the aggregation of α-syn [190]. First, they used a thioflavin T fluorescence assay and found that GQDs significantly reduce the levels of α-syn (Fig. 11a). Moreover, after incubation with GQDs in culture medium, they found α-syn fibrils to be decomposed (Fig. 11b). It appeared that GQDs bind to the existing α-syn fibrils through hydrophobic interactions and thereby promote their degradation. For in vivo experiments, Kim et al*.* simulated the PD pathological state in the striatum (Fig. 11c). After a period of treatment, they found that the striatum and SN of mice that received only PFF injections showed a significant reduction in TH^+^ neurons, while the number of TH^+^ neurons in the GQDs treatment group was significantly higher (Fig. 11d, e). Further, GQD-treated mice showed significant improvements in behavioral tests for PD symptoms (Fig. 11f). Importantly, GQD processing reduced the distribution of α-syn in the brain (Fig. 11g). Overall, these results demonstrate that GQDs can inhibit α-syn-induced neurotoxicity. Moreover, molecular simulation studies have revealed that negatively charged GQDs, which have a large number of hydroxyl groups on their surface, can easily bind to the positively charged N-terminal of α-syn, inhibiting the formation of α-syn monomers. Likewise, GQDs can also bind to the N-terminal of the fibrils, which changes the van der Waals energy in the system, leading to a degradation of the fibrils. In another study, Hajipour et al*.* explored the mechanism underlying the interaction between the α-syn protein and graphene and superparamagnetic iron oxide NPs [193]. They found that both materials bind to the N-terminal of α-syn and its fibrils through electrostatic force. Therefore, it is reasonable to believe that nanomaterials with negatively charged surfaces, and especially those with a larger specific surface area, can effectively inhibit the PD symptoms caused by abnormal α-syn aggregation.

However, recent reports indicate that inhibiting the aggregation of pathogenic proteins may not result in clinical effects similar to those observed in preclinical studies [208]. Therefore, the aforementioned excellent anti-PD efficacy in the PD animal model still needs to be validated in patients. Importantly, the co-existence of abnormal amyloid and Tau protein aggregation in AD could explain the different results in clinical settings. In contrast, the lone target protein in PD is well known; hence, the strategy can be easily improved to ensure appropriate clinical application. It should be noted that in addition to inhibiting its aggregation, considering α-syn as a therapeutic target can also reduce its production, accelerate intracellular degradation, and inhibit the cellular uptake of α-syn oligomers. Gene delivery via nanocarriers is widely used to inhibit the production of α-syn. However, this method also has drawbacks because the excessive inhibition of α-syn production may affect the transport of synaptic vesicles [209]. However, no studies have used nanotechnology to accelerate intracellular degradation and inhibit the cellular uptake of α-syn. In addition, the biggest challenge in the field at present is the insufficient understanding of α-syn. First, the specific physiological functions of α-syn have not been clarified; second, α-syn has many forms, including monomers, misfolded monomers, oligomers, fibers, and Lewy bodies. It is currently clear that the neurotoxicity of α-syn is only related to its oligomers and higher-order forms, and its specific structure still needs to be explored further. Moreover, it is not clear how neurotoxic α-syn undergoes intercellular transmission. This information could be helpful for designing α-syn targeted drugs, and will be of great significance for the treatment of PD.

### Regeneration of Dopaminergic Neurons

For a long time, PD treatment was primarily focused on slowing down the death of neuronal cells or limiting disease progression. Recently, the regeneration of neurons has emerged as a new strategy [210, 211]. The loss of dopaminergic neurons in PD, which causes dyskinesia and non-motor dysfunction, is the main pathological change aggravating PD symptoms. The massive loss of dopaminergic neurons located in the substantia nigra can directly reduce the release of dopamine in the striatum, impairing motor function, which is the core pathological feature of PD. Therefore, restoring the vitality of dopaminergic neurons can attenuate the Parkinsonism induced by dopamine deficiency, and promoting their regeneration is undoubtedly the most promising PD treatment strategy.

**Fig. 12:**
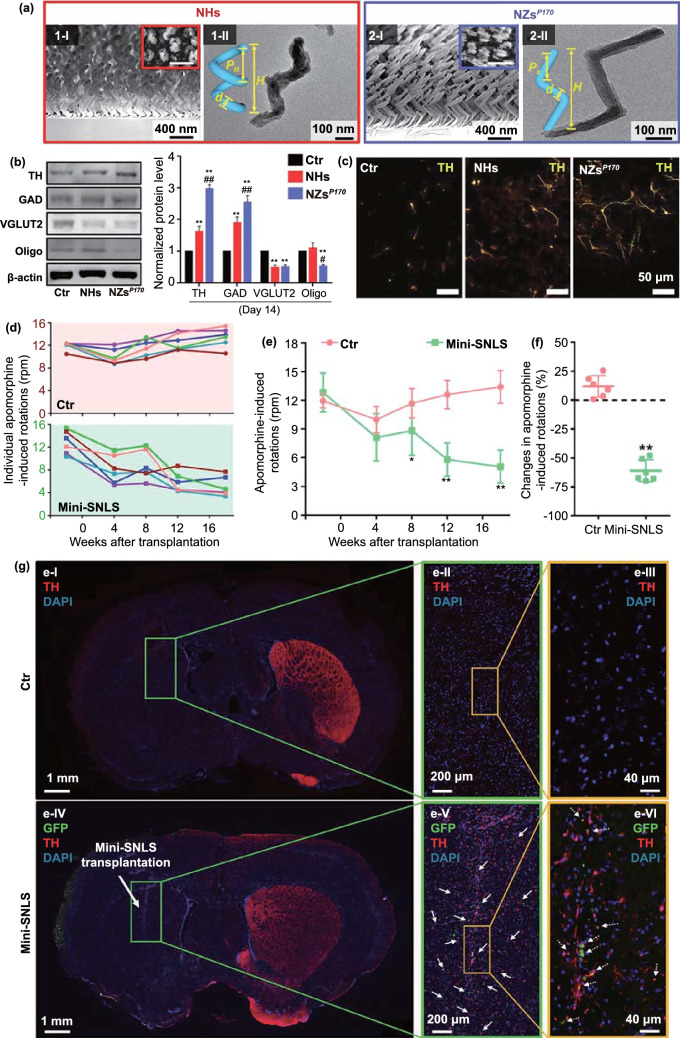
Example of dopaminergic neuron regeneration via stem cells. **a** Glancing angle deposition (GLAD) of the silica iSECnMs sculptured into (1) NHs and (2) NZsP170: I) scanning electron microscopy (SEM) cross-sectional images (insets: SEM top-down images); II) transmission electron microscopy images of individual nanostructures (insets: diverse structural schemes of NHs and NZs). **b** Specific differentiation of NSCs on different substrates. Western blotting was used to evaluate the expression of various protein markers of differentiation (TH, GAD, VGLUT2, and Oligo) on day 14. **c** Immunocytochemical analysis of dopaminergic (DA) neurons induced on different substrates, with representative images of TH staining (yellow). **d** Individual apomorphine-induced rotations in rats without (control rats, Ctr; pink background) and with transplanted mini-SNLSs (mini-SNLS rats; green background) as a function of time. **e** Statistical analysis of apomorphine-induced rotations in the Ctr (pink) and mini-SNLS (green) rats. **f** Changes in the apomorphine-induced rotations in the Ctr (pink) and mini-SNLS (green) rats in the 18th week post-transplantation; **g** Immunohistochemical analysis of brain coronal sections in I–III) Ctr rats and IV–VI) mini-SNLS rats in the 18^th^ week post-transplantation: TH (red), GFP (green), and DAPI (blue). The boxed area in each image is magnified on the right. Grafted cells (solid arrows in panel [e-V]) and double-labeled cells (dotted arrows in panel [e-VI]) were widely distributed around the primary transplantation site. Reproduced from Ref. [196]

The direct implantation of exogenous cells is challenging owing to natural rejection. Interestingly, a recent study showed that the direct implantation of organ tissue analogs can be used to effectively regenerate brain neurons [212]. Based on this concept, Zhang et al*.* attempted to induce differentiation in stem cell analogs of brain tissue using the biological activity of nanomaterials [196]. Through glancing angle deposition, they developed silica-based organic sculptured extracellular nanomatrices (iSECnMs) that were carved into helices (nanohelices, NHs) and zigzags (nanozigzags, NZs) (Fig. 12a). The incubation of neural stem cells (NSCs) in a solution containing NHs and NZs for 14 days led to their differentiation. Overall, both NHs and NZs increased the expression of TH and glutamic acid decarboxylase (GAD), a marker of GABAergic neurons, which can strengthen the functions of dopaminergic neurons in the SN, while inhibiting VGLUT2 (a glutamatergic neuron marker) and Oligo (an oligodendrocyte marker) (Fig. 12b-c). Compared to NHs, NZs performed better as their unique groove-like topography improved cell survival. Therefore, NZs could be used to directly stimulate the generation of SN-like structures (mini-SNLSs). The implantation of mini-SNLSs in 6-OHDA-induced rat models of PD significantly reduced dyskinesia (Fig. 12d-f). Notably, after the 24^th^ week of 6-OHDA injection, TH^+^ neurons were completely undetectable in the left brain of the PD model rats. In contrast, after 18 weeks of mini-SNLS implantation, the presence of a large number of TH^+^ neurons was evident (Fig. 12g). Though this method is highly promising, it has the drawbacks of complicated surgery and low safety, and a minimally invasive/non-invasive method is required to increase patient compliance.

**Fig. 13:**
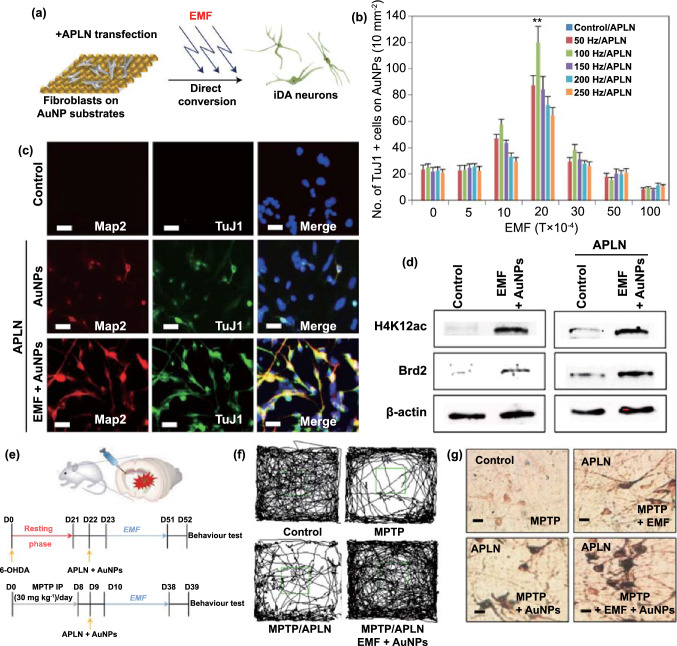
Example of dopaminergic neuron regeneration via fibroblasts. **a** Schematic showing the process of direct lineage reprogramming of fibroblasts into Ida neurons using EMF-induced AuNP magnetization. **b** Number of TuJ1^+^ cells generated on magnetized AuNPs under different intensities and frequencies of EMF. **c** Immunostaining for the mature neuron markers MAP2 and TuJ1 in cells grown on a control AuNP substrate and magnetized AuNP substrate after exposure to 2 × 10^–3^ T/100 Hz EMF. **d** Western blotting for H4K12ac and Brd2 in control fibroblasts and EMF-exposed fibroblasts with and without the reprogramming factor APLN. **e** Schematic of in vivo direct lineage reprogramming using EMF-induced magnetized AuNPs in an MPTP- or 6-OHDA-induced PD mouse model. **f** Track sheets show the alteration in locomotory function in the MPTP mouse model (control, MPTP, MPTP + APLN and MPTP + APLN + EMF + AuNPs). **g** Representative image of DAB-TH staining in EMF-induced Ida neurons in the striatum from the control, EMF only, AuNPs only, and EMF + AuNPs groups. Adapted with permission from Ref. [195]. Copyright 2017 Nature Publishing Group

Both immune rejection and operation difficulties can be overcome by differentiating endogenous cells into new dopaminergic neurons, and this is therefore a much better choice for clinical application. Earlier, it was believed that only stem cells can differentiate into neurons, but a recent report showed that even astrocytes can transform into neurons [213], highlighting the great potential of the “neuron regeneration” strategy in neurodegenerative diseases. Since this method involves the knocking out of the RNA-binding protein PTB, there are certain technical challenges involved. In their study, Yoo et al*.* demonstrated that in the presence of an external electromagnetic field (EMF), gold NPs (AuNPs) can induce dopaminergic neuron differentiation in fibroblasts containing dopamine lineage transcription factors or regeneration factors (APLN) (Fig. 13a), greatly reducing technical challenges [195]. To determine the most appropriate EMF intensity, they first measured the efficiency of tail-up fibroblast (TTF) conversion into DA neurons under different EMF intensities using TuJ1^+^ as a marker. The EMF intensity of 2 × 10^–3^ T/100 Hz showed the highest conversion rate (Fig. 13b) and was used for further in vitro and in vivo experiments. Immunostaining revealed that under the combined action of AuNPs and EMF, somatic fibroblasts showed a significant increase in the intracellular content of TuJ1 and Map2 (another DA neuron marker) (Fig. 13c), suggesting their transformation into DA neurons. Under this condition, western blotting revealed that multiple proteins, most significantly *Brd2*, showed significant changes in expression (Fig. 13D). Importantly, *Brd2* expression and the EMF + AuNP-induced cell transformation were positively co-related. Furthermore, in vivo experiments revealed that EMF + AuNPs significantly improved behavioral parameters in PD model mice by increasing the number of DA neurons in the striatum as shown by immunohistochemistry (Fig. 13e-g).

Nevertheless, there are certain challenges to this “neuron regeneration” strategy as well. In particular, the length of the treatment cycles and the efficacy need to be improved in the future. Moreover, there are few target cells that can successfully achieve in vivo neuronal differentiation. Thus, to ensure the addressal of these concerns and ensure the overall safety of the procedure, future research is warranted in this direction.

## Patents

In recent years, basic nanotechnology research aimed at improving PD treatment has developed rapidly. Most studies have focused on safety, precision, efficiency, controllability, and prospective clinical application. To provide an understanding of the current and future development trends in this area, recent patent applications in this field are listed in Table 4.

**Table 4:** Patents related to nanotechnology-based PD treatment filed in recent years

Patent/application number	Patent title	Nanoplatform	Assignee	Filing year	Status
WO2011119588A1	Intravenous curcumin and derivatives for treatment of neurodegenerative and stress disorders	Liposome	SIGNPATH PHARMA INC, US	2011	Filed
WO2013134777A1	Methods for delivery to the central nervous system of nucleic acid nanoparticles to treat central nervous system disorders	Nucleic acid nanoparticle	Northeastern University (US). Copernicus Therapeutics, INC, US	2013	Granted
US20130129815A1	Liposomal formulation for oral administration of glutathione (reduced) via gel capsule	Liposome encapsulation in a gel cap	Your Energy Systems, LLC Guilford Frederick Timothy Keller Brian C, US	2013	Filed
WO2014037881A1	Compositions and methods for Parkinson’s disease treatment by BDNF-FLGA gene transfer through neurotensin polyplex to nigral dopamine neurons	NTS-polyplex	CENTRO DE INVESTIGACIÓN Y DE ESTUDIOS AVANZADOS DEL INSTITUTO POLITÉCNICO NACIONAL, MX	2013	Filed
WO2014153160A2	Method of enhancing the biodistribution and tissue targeting properties of therapeutic CeO_2_ particles via nano-encapsulation and coating	Nano-encapsulation CeO_2_ particle	PEROXYIUM, INC., DELAWARE CORP, US	2014	Filed
WO2015087266A2	Methods for treating Parkinson’s disease by an agonist D3 and BDNF transfection-combination therapy	NTS-polyplex	CENTRO DE INVESTIGACIÓN Y DE ESTUDIOS AVANZADOS DEL INSTITUTO POLITÉCNICO NACIONAL, MX	2014	Filed
US20150335764A1	Composition and methods for Parkinson’s disease treatment by BDNF-FLAG genet transfer through neurotensin polyplex to nigral dopamine neuro	NTS-polyplex	CENTRO DE INVESTIGACIÓN Y DE ESTUDIOS AVANZADOS DEL INSTITUTO POLITÉCNICO NACIONAL, MX	2015	Filed
US20160279263A1	Drug delivery compositions and methods targeting P-glycoprotein	PEG-modified nanosphere	Cornell University, Ithaca, NY (US)	2013	Filed
US20160271269A1	Peptidic nanodelivery composition targeting two receptors	Polypeptide	Seyed Moien MOGHIMI, København Ø (DK); Linping WU, Søborg (DK); Davoud AHMADVAND, Søborg (DK); Ladan HAMIFARR, Fredriksberg (DK); Thomas Lars ANDRESEN Vanløse (DK)	2014	Filed
US20170252486A1	Biomaterials for neuronal implants and use of said biomaterials in the diagnosis and therapy of neuronal disease	Stochastic nanoroughness	Albert-Ludwigs-Universitat Freiburg, Freiburg, DE	2015	Filed
WO2018187240A1	Deformable nanoscale vehicles [DNVS] for trans-blood brain barrier, trans-mucosal, and transdermal drug delivery	Nanoscale vehicles [DNVS]	The Regents of the University of California, US	2018	Filed
WO2018028117A1	Scorpion venom heat-resistant synthesized peptide and uses thereof	Polypeptide	UNIV DALIAN MEDICAL, China	2016	Filed
US20190351231A1	Nanoparticles for use for treating a neuronal disorder	Nanoparticle	NANOBIOTIX, PARIS (FR)	2017	Filed
US20190351057A1	Coated nanoparticles for use for modulating electrical polarization of neurons	Coated silver nanoparticle	NANOBIOTIX, PARIS (FR)	2017	Filed
US20190151242A1	Liposome composition for relieving symptoms of Parkinson’s disease and Alzheimer’s disease	Liposome	Jeng-Fong CHANG, Taibei (TW); Hui KUNG, Taibei City (TW)	2018	Filed
US20190029970A1	Fatty acid conjugated nanoparticles and use thereof	Polymeric nanoparticle	The Chinese University of Hong Kong, Shatin (CN)	2018	Filed
WO2019018928A1	Nanoscale optoregulation of neural stem cell differentiation	Gold nanoparticle	SANATI NEZHAD, Amir [IR/CA]; HASNI-SADRABADI, Mohammad Mahdi [IR/US]; MOSHAVERINIA, Alireza [IR/US]; JACOB, Karl I [US/US]; HASSAN-POUR-TAMRIN, Sara [IR/CA]	2017	Filed
US20200093769A1	Nanoparticle compositions, methods of fabrication, and use for drug delivery	Polymeric nanoparticle	DIGNITY HEALTH, Phoenix, AZ (US)	2018	Filed
CN201410289953.4	Multifunctional nano-biological transfection reagent with gene therapy effect of Parkinson’s disease, preparation method, and application thereof	Fe_3_O_4_ nanoparticles wrapped in hydrogel	South China Normal University, China	2014	Granted
CN201410507653.9	BDNF gene-polycation nanoparticle complex and preparation method and application thereof	BDNF gene-polycation nanoparticle complex	Xinhua Hospital, Shanghai Jiaotong University School of Medicine, China	2014	Filed
CN201410507664.7	GDNF gene-polycation nanoparticle complex and preparation method and application thereof	GDNF gene-polycation nanoparticle complex	Xinhua Hospital, Shanghai Jiaotong University School of Medicine, China	2014	Filed
CN201510060179.4	Gold nanoparticle compound with function of inhibiting apoptosis of neurons and application thereof	Gold nanoparticle	South China Normal University, China	2015	Granted
CN201611136048.0	Preparation method of dual drug-loaded liposomes for treating Parkinson’s disease	Liposome	Wenzhou Traditional Chinese Medicine Hospital, China	2016	Filed
CN201710561342.4	Synthesis and application of gene-targeted degradable nano-magnesium oxide metal complex	Nano-magnesium oxide metal colloid	South China Normal University, China	2017	Granted
CN201710561360.2	Acteoside entrapping mPEG-PLA nanomicelle composite as well as synthesis method and application thereof	Acteoside mPEG-PLA nanomicelle complex	South China Normal University, China	2017	Granted
CN201710425636.4	Long-acting sustained-release preparation of anti-Parkinson’s disease medicine and preparation method thereof	Microspheres	Guangzhou Diqi Pharmaceutical Technology Co., Ltd., China	2017	Granted
CN201710597787.8	Application of substance containing gold nanoclusters in preparation of drugs for preventing and treating Parkinson’s disease	Gold nanocluster	Shenzhen Shenjian Pharmaceutical Technology Co., Ltd., China	2017	Filed
CN201710801721.6	A kind of EGCG perfume medicine for treating Parkinson’s disease and its preparation method	Fe_3_O_4_ nanoparticle	Institute of Process Engineering, Chinese Academy of Sciences, China	2017	Granted
CN201810348534.1	A brain-targeted nanoformulation and its carrier for realizing combined administration of levodopa and curcumin to treat Parkinson’s disease	Lipid-encapsulated mesoporous silica nanoparticle	Institute of Pharmaceutical Biotechnology, Chinese Academy of Sciences, China	2018	Granted
CN201811316786.2	Bio-nanomaterial complex and its synthesis method and application	Copper sulfide nanoparticle	East China Normal University, China	2018	Filed
CN201811179924.7	A liposome-modified gold nanoparticle complex and its application in treating Parkinson’s disease	Liposome-modified gold nanoparticle complex	South China Normal University, China	2018	Filed
CN201811526887.2	Application of water-soluble fullerene structure in preparing medicine for treating Parkinson’s disease	Fullerene	Institute of Chemistry, Chinese Academy of Sciences, China	2018	Filed
CN201910359952.5	Application of ApoD in preparation of Parkinson’s disease medicine, ApoD preparation and preparation method thereof	Liposome	Qingdao University, China	2019	Granted
CN201910039113.5	Copper nanoclusters, thymine-modified hyaluronic acid, and polymeric copper clusters, preparation method, and application thereof	Copper nanocluster	Shenzhen Guangxing Scientific Research Co., Ltd., China	2019	Filed
CN202010142569.7	Brain-targeting nanoformulation and application of levodopa and curcumin in preparing medicine for treating Parkinson’s disease	Modified liposome-encapsulated mesoporous silica nanoparticle	Institute of Pharmaceutical Biotechnology, Chinese Academy of Medical Sciences, China	2018	Filed
CN202010103342.1	Cell membrane nanovesicles and preparation method thereof	Cell membrane nanovesicle	The First Affiliated Hospital of Dalian Medical University, China	2020	Filed

In the early days of development, the role of nanomaterials was limited to drug or gene delivery, and nanoparticles only carried one type of anti-PD drug. Recently, trials for a combination of multiple drugs have also been initiated. For example, CN202010142569.7 developed a brain-targeted drug delivery system composed of a lipid bilayer modified with cell-penetrating peptides and lactoferrin as the outer membrane enclosing mesoporous silica NPs carrying levodopa and curcumin. This advanced nanocarrier allows the enrichment of levodopa and curcumin in the brain without allowing aggregation in other organs and creating adverse effects. Moreover, the synergistic anti-PD effect of levodopa, which reduces dyskinesia, and curcumin, which exerts a neuroprotective effect, became feasible.

The patients filed over recent years also suggest that PD treatment strategies are no longer limited to simple chemotherapy or gene therapy but are instead being diversified. For example, CN201811526887.2 proposes the direct use of nanomaterials for antioxidant anti-PD effects. The continuous intraperitoneal injection of fullerenes, which have ROS-scavenging capability, into MPTP-induced mouse models of PD is known to improve dopamine content and the number of TH^+^ neurons in the brain. The copper nanocluster described in CN201910039113.5 has similar functions. Moreover, other patents describe the neuron regeneration strategy for PD treatment. CN202010103342.1 involves the use of a biomimetic drug for nerve repair. The repeated squeezing of RAW264.7 or stem cells facilitates the generation of 100–200-nm nanovesicles that effectively inhibit MPP^+^-induced cytotoxicity in midbrain neuron cells.

Briefly, these patient-related studies have shown excellent anti-PD efficacy in animals, and the field is expected to diversify, resulting in an increase in clinical application in the near future. However, these patents expose the huge gap between current research and actual applications. Outside of laboratory research, only few patents improve PD symptoms by eliminating excessive ROS, inhibiting the aggregation of disease-causing proteins, and nerve regeneration. Moreover, the clinical translation in this field is suboptimal, mainly for two reasons. First, although preclinical studies examine the safety of most anti-PD nanomedicines in animals based on diverse indicators such as cytotoxicity, H&E staining, pharmacokinetic testing, tissue distribution, blood routine, and liver and kidney functions, there are large differences between animals and humans. The safety of these nanomedicines in humans needs rigorous evaluation. Second, the preparation of nanomedicines requires technical precision, making reproduction in industrial settings difficult. Presently, the progress of nanotechnology-based PD treatment is also limited by clinical trial approvals for ethical reasons. Overall, for the rapid transition of nanotechnology-based anti-PD drugs from the preclinical to clinical stage, huge efforts from all participants will be required.

## Conclusion and Outlook

Aging will cause an increase in the number of individuals with PD. Since PD cannot be cured currently, the development of new anti-PD drugs is required. Nanotechnology-based anti-PD drugs can provide precise and well-controlled brain-targeted delivery and therefore have been receiving extensive attention in recent years. In this review, we highlight various strategies for crossing the BBB and shed light on the nanomaterials with anti-PD nano-biological effects to provide a new reference for their clinical application.

The BBB makes it difficult for anti-PD drugs to enter the brain parenchyma, but there are various receptor- and carrier-mediated pathways that allow the movement of molecules as observed in the simple diffusion of lipophilic substances. However, common anti-PD nanomedicines have focused on brain-targeted delivery and ignored the drug distribution problem in the brain parenchyma. At present, only a few ligands, such as RVG29, can provide secondary targeting, but these too face the challenges observed with the RMT strategy. In this review, other strategies, which are not dependent on physiological transport pathways across the BBB, have also been discussed. These include nasal administration to bypass the BBB, biomimetic drugs, magnetic mediation, and enhancement of BBB permeability through photothermal action and ultrasound. Though these techniques have been proven effective, their brain-targeting efficiency and safety need further improvement. The effects of photothermal action and confocal ultrasound on BBB permeability are both reversible and safe. Each BBB-crossing strategy has its unique advantages and disadvantages.

In sum, BBB-crossing strategies based on biological effects (including transcellular lipophilic transport, carrier-mediated transport, receptor-mediated transport, nasal delivery, and biomimetic drug delivery) can safely and effectively increase the concentration of drugs in the brain. However, the concentration of these drugs in other organs, especially the liver and kidney, becomes much greater than that in the brain, indicating that the brain delivery efficiency of these strategies needs to be further improved. Although nasal delivery can prevent this problem, the clinical translation of this strategy can only be achieved once the following two problems are solved. First, at present, syringes suitable for human nasal delivery are still unavailable. Second, the drug needs to be retained in the nasal cavity without affecting normal physiological activities, so as to prevent the drug from flowing out of the nasal cavity without being absorbed. In addition, biomimetic drug delivery to the brain is mediated by membrane proteins, which are easily degraded during the preparation progress. Hence, obtaining biomimetic carriers without protein loss or with less protein loss is still a huge challenge. Further, although spatiotemporally controlled brain-targeted drug delivery can be achieved with physical strategies, safety remains the most important concern preventing clinical translation. Excessive external stimulation can not only damage the BBB but may also cause new brain diseases. In particular, magnetic delivery based on IONPs involves the use of iron particles, which can induce the Fenton reaction and thereby aggravate Parkinsonism. To avoid the shortcomings of single strategies and achieve secondary targeting, a combination of multiple strategies is necessary and will be the mainstay of brain disease research in the future.

Three new treatment strategies for the “incurable” condition PD have been proposed in recent years. These include removal of excess ROS, inhibition of the abnormal aggregation of the pathogenic protein α-syn, and neuron regeneration. Moreover, advanced nanocarriers providing synergistic effects can significantly enhance anti-PD efficacy. However, existing nano-bio effects for PD therapy are still not sufficient. Most nanomaterials possess nano-immune interfaces, which allow them to regulate the immune microenvironment in the brain. To improve PD treatment, a suitable nano-bio effect that causes the induction of the M2 phenotype in microglia or astrocytes, resulting in anti-inflammatory effects, is more favorable. More importantly, since nanoplatforms enter the systemic circulation, especially through the oral route, they also partially aggregate in the intestinal tract. Given the discovery of the “brain–gut axis” in recent years, it is also worthy to examine whether these nanoplatforms can shift the gastrointestinal microbiome to a condition more favorable for PD treatment. This may open up new avenues for PD theranostics.

It is worth noting that although these strategies have shown excellent anti-PD effects in animal models, current methods for establishing PD models in animals, such as MPTP and 6-OHDA administration, cannot completely simulate PD. Hence, preclinical data need to be further tested. Unfortunately, the patents summarized in the present review reveal the huge gap between preclinical findings and clinical applications.

As a disease with multiple causes and many pathological and risk factors, PD will likely be difficult to cure using single-target therapy. A “cocktail” therapy that provides multi-target effects while alleviating oxidative stress, stimulating neuroinflammation, removing neurotoxic α-syn, promoting the regeneration of dopaminergic neurons, and regulating intestinal flora may be a more appropriate PD treatment strategy. Although the design of multi-target drugs is difficult, nanotechnology can help in overcoming such problems, bringing new hope for such multi-target “cocktail” therapy. We hope that this review can provide more inspiration for additional research in this field. We also believe that by relying on nanotechnology, humans will eventually defeat PD.
